# Societal perspective on access to publicly subsidised medicines: A cross sectional survey of 3080 adults in Australia

**DOI:** 10.1371/journal.pone.0172971

**Published:** 2017-03-01

**Authors:** Lesley Chim, Glenn Salkeld, Patrick Kelly, Wendy Lipworth, Dyfrig A. Hughes, Martin R. Stockler

**Affiliations:** 1 Sydney School of Public Health, The University of Sydney, Sydney, NSW, Australia; 2 Alexion Pharmaceuticals, Sydney, NSW, Australia; 3 Faculty of Social Sciences, University of Wollongong, Wollongong, NSW, Australia; 4 Centre for Values, Ethics and the Law in Medicine, The University of Sydney, Sydney, NSW, Australia; 5 Centre for Health Economics and Medicines Evaluation, Bangor Institute for Health & Medical Research, Bangor University, Bangor, Wales, United Kingdom; 6 National Health and Medical Research Council (NHMRC) Clinical Trials Centre, The University of Sydney, Sydney, NSW, Australia; 7 Concord Cancer Centre–Concord Hospital, Sydney, NSW, Australia; 8 Chris O’Brien Lifehouse, Sydney, NSW, Australia; Jagiellonian University, POLAND

## Abstract

**Background:**

Around the world government agencies responsible for the selection and reimbursement of prescribed medicines and other health technologies are considering how best to bring community preferences into their decision making. In particular, community views about the distribution or equity of funding across the population. These official committees and agencies often have access to the best available and latest evidence on clinical effectiveness, safety and cost from large clinical trials and population-based studies. All too often they do not have access to high quality evidence about community views. We therefore, conducted a large and representative population-based survey in Australia to determine what community members think about the factors that do and should influence government spending on prescribed medicines.

**Methods:**

A choice-based survey was designed to elicit the importance of individual criteria when considering the equity of government spending on prescribed medicines. A representative sample of 3080 adult Australians completed the survey by allocating a hypothetical budget to different combinations of money spent on two patient populations. Societal preferences were inferred from absolute majority responses i.e. populations with more than 50% of respondents’ allocation for a particular allocation criterion.

**Results:**

This study shows that, all else being equal, severity of disease, diseases for which there is no alternative treatment available on the government formulary, diseases that affect patients who are not financially well off, and life-style unrelated diseases are supported by the public as resource allocation criteria. Where ‘all else is not equal’, participants allocated more resources to the patient population that gained considerable improvement in health and fewer resources to those that gained little improvement in health. This result held under all scenarios except for ‘end-of-life treatments’.

Responses to cost (and corresponding number of patients treated) trade-off scenarios indicated a significant reduction in the proportion of respondents choosing to divide resources equally and a shift in preference towards devoting resources to the population that were more costly to treat for all criteria with the exception of severity of disease.

**Conclusions:**

The general public have clear views on what’s fair in terms of government spending on prescribed medicines. In addition to supporting the application of the ‘rule of rescue’, important considerations for government spending included the severity of disease being treated, diseases for which there is no alternative treatment available on the government formulary, diseases that affect patients who are not financially well off and life-style unrelated diseases. This study shows that the general public are willing to share their views on what constitutes an equitable allocation of the government’s drug budget. The challenge remains to how best to consider those views alongside clinical and economic considerations.

## Introduction

Since the 1940s, the Pharmaceutical Benefit Scheme (PBS), Australia’s national formulary for publicly subsidised medicines, has endeavoured to provide all citizens and residents with timely and equitable access to affordable, safe and effective medicines. While most PBS medicines are dispensed by community pharmacies and used by patients at home, some medicines are supplied through different distribution arrangements (Section 100 programs) e.g. distribution from hospital outpatient departments [1, 2].

The process for listing medicines on the PBS is underpinned by legislation that requires an independent expert committee, the Pharmaceutical Benefits Advisory Committee (PBAC), to consider clinical effectiveness, safety and cost-effectiveness relative to existing therapies [3] prior to making a recommendation to the Minister of Health for listing a drug on the PBS.

Evidence suggests that the PBAC has been broadly consistent in its use of economic efficiency as a key criterion for decision making. George et al [4], for example, analysed PBAC recommendations for the listing of drugs on the Australian PBS between 1991 and 1996, and demonstrated that drugs with lower cost-effectiveness ratios had a higher chance of gaining a positive recommendation and subsidy. However, cost-effectiveness was not the only factor determining the PBAC’s recommendation. Other factors such as clinical need for the product and lack, or inadequacy, of alternative treatments also figured in the PBAC recommendations [4]. Harris et al [5] analysed PBAC recommendations between 1994 and 2004 and demonstrated that clinical significance, cost effectiveness, cost to the government and severity of disease were all significant influences on PBAC recommendations and concluded that there was no evidence of a fixed threshold for the value of a life year or a quality adjusted life year (QALY) [5].

While such retrospective analyses are important, they do not tell us much about societal views on funding new medicines with respect to distributional equity. To answer this question, we need to take into consideration societal views on the selection and reimbursement of prescribed medicines. One area that is particularly in need of societal input is that of high cost anti-cancer medicines given the rapid emergence of new, expensive and innovative medicines [6] as well as an increasing prevalence of cancer [7].

While there does not appear to be evidence that cancer patients are at a systematic disadvantage when it comes to PBAC recommendations [8], rejections of new anti-cancer medicines have been contentious, and often result in public indignation and organised campaigns to lobby for better drug access and coverage [9, 10]. This kind of dissent suggests that there remains a significant gap between policy makers and the public when it comes to assessments of the value of new anti-cancer drugs [11]. A recent Senate Inquiry conducted by the Australian government focused on examining timely access and affordability of anti-cancer drugs, and how this impacts upon the quality of cancer care [12]. The resulting Senate Report concluded that the Government needs to undertake a “comprehensive review” of its processes for funding anti-cancer medicines, including considerations of “managed access” programs and “more flexible evidential requirements”. However the report had little to say about how to ensure that the system remains robust and sustainable [12].

Given the number of new high-cost anti-cancer drugs expected to be marketed in coming years, and limits to the amount of money that governments are willing to spend on medicines, reimbursement will continue to be a key challenge for decision makers in all healthcare systems [13, 14]. Bodies such as the PBAC will need to continually weigh up competing ethical, clinical, epistemic and economic considerations. One approach to assisting policymakers in striking the right balances and compromises is to ask the public who should have access to subsidised medicines and what decision characteristics (factors) should be considered when assessing overall societal value of a new medicine [15].

Previous studies have elicited the general public’s preferences for access to publicly subsidised medicines. For example, a pilot study by Whitty and colleagues [16] found that the public (n = 161) and individual decision makers involved in the PBAC process (n = 11) preferred to treat those with severe illness. More recently, Linley et al [17] conducted a survey to elicit general public views about the criteria used by the National Institute of Health and Care Excellence (NICE) for accepting higher incremental cost effectiveness ratios for some medicines over others, and about the introduction of the Cancer Drugs Fund (CDF) in England. Linley et al [17] showed that the general public supported trade-offs in equity and efficiency in the allocation of health care resources. However, it is not clear if UK societal preferences reflect preferences of the Australian public for pharmaceutical funding decisions. Further, studies have been undertaken among different stakeholder groups (including payers, government agencies, patients, healthcare professionals, academia or the general public) in a different context based on a multi-attribute approach to identify criteria or factors that could influence healthcare resources allocation [18, 19]. Vogler et al [18] elicited preferences about policy objectives while the study by Tordrup et al [19] focussed on the policy options for future health system financing.

The aim of this study was to explore preferences of the Australian public when it comes to government spending on medicines.

## Methods

### Questionnaire design

We conducted a survey of 3080 members of the Australian general public to identify criteria that are important to the public when assessing new medicines for PBS spending. The on-line survey was based on a recent preference survey conducted by Linley et al in the UK [17] and adapted to issues relevant to the Australian PBS. Respondents were presented with two hypothetical patient groups and 12 different scenarios where the only difference between each scenario was a single criterion. Those criteria included: severity of disease, availability of an alternative treatment, innovation in drug mechanism, carer burden, disadvantaged populations (patients who are not financially well off), age (children), life expectancy, disease type (specifically cancer), prevalence of disease, cost, return to work benefits, life-style related disease. A summary of the 12 allocation criteria and trade-off scenarios explored in this study are presented in **Table 1**. Each of the 12 allocation criteria is known to be considered by the Australian drug selection committee (PBAC) when making a recommendation for listing on the PBS or supported by the published literature as important criteria for resource allocation decisions [16, 17, 20–22].

**Table 1:** Allocation criteria explored including cost and benefit trade-off scenarios.

Allocation criteria explored	Baseline: All else being equal (equal treatment costs and effectiveness)	Benefit trade-off scenario	Cost trade-off scenario
Severity of disease^**1**^	Should more PBS money go to patients with severe health problems (Pop 1) compared to those with moderate health problems (Pop 2)?	Trade-off scenario explored smaller health gain for severe disease compared with moderate disease	Trade-off scenario explored higher costs of treatment for severe disease compared with moderate disease
Availability of alternative treatment option as proxy for unmet need^**1**^	Should more PBS money go to patients for whom there are no alternative treatments available on the PBS (Pop 1) compared to those for whom there are several alternative treatments already available on the PBS (Pop 2)?	Trade-off scenario explored smaller health gain for the disease with no alternative treatment available on the PBS compared with the disease with several alternative treatments already available on the PBS	Trade-off scenario explored higher costs of treatment for the disease with no alternative treatment available on the PBS compared with the disease with several alternative treatments already available on the PBS
Innovative medicines	Should more PBS money go to treatments that work in new ways (Pop 1) compared to treatments that work the same way as existing treatments (Pop 2)?	Trade-off scenario explored smaller health gain for medicine that has an innovative mechanism of action compared with medicine that works in the same way as other existing medicines	Trade-off scenario explored higher costs of treatment for medicine that has an innovative mechanism of action compared with medicine that works in the same way as other existing medicines
Care burden/wider societal benefit^**1**^	Should more PBS money go to patients who have to rely on carers for their day-to-day needs (Pop 1) compared to those who do not have to rely on carers (Pop 2)?	Trade-off scenario explored smaller health gain for disease that causes patients to be dependent on carers (e.g. family members) for day-to- day needs compared with the disease that allows patients to remain independent	Trade-off scenario explored higher costs of treatment for disease that causes patients to be dependent on carers (e.g. family members) for day-to- day needs compared with the disease that allows patients to remain independent
Disadvantaged populations^**1**^	Should more PBS money go to patients who are not financially well-off (Pop 1) compared to those who are financially well-off (Pop 2)?	Trade-off scenario explored smaller health gain for disease that typically affects patients who are not financially well-off (e.g. patients from low income families) compared with the disease that typically affects patients who are financially well-off	Trade-off scenario explored higher costs of treatment for disease that typically affects patients who are not financially well-off (e.g. patients from low income families) compared with the disease that typically affects patients who are financially well-off
Children^**1**^	Should more PBS money go to treating children (Pop 1) compared to treating adults (Pop 2)?	Trade-off scenario explored smaller health gain for children compared with adults	Trade-off scenario explored higher costs of treatment for children compared with adults
Life expectancy/end of life treatments^**1**^	Should more PBS money go to patients who would die within 18 months without treatment (Pop 1) compared patients who would die within 60 months without treatment (Pop 2)?	Trade-off scenario explored smaller health gain (life extension of 3 months vs. 6 months) for patients with a life expectancy of 18 months compared with 60 months without treatment	Trade-off scenario explored higher costs of treatment for patients with a life expectancy of 18 months compared with 60 months without treatment
Cancer treatments^**1**^	Should more PBS money go to patients who have cancer (Pop 1) compared to patients with a non-cancer disease (Pop 2)?	Trade-off scenario explored smaller health gain for patients with cancer compared with non-cancer disease	Trade-off scenario explored higher costs of treatment for patients with cancer compared with non-cancer disease
Rare disease therapies^**1**^	Should more PBS money go to patients with rare diseases (Pop 1) compared to those with common diseases (Pop 2)?	Trade-off scenario explored smaller health gain for patients with a rare disease compared with common disease	Trade-off scenario explored higher costs of treatment for patients with a rare disease compared with common disease
Cost to the PBS and savings to patients	Should more PBS money go to patients whose out of pocket costs without PBS subsidy would be high (Pop 1) compared to those whose out of pocket costs would be low (Pop 2)?	Trade-off scenario explored smaller health gain for patients with a disease that costs the PBS $5000/saves patients $4960 per month compared with the disease that costs the PBS $100/saves patients $60 per month	Trade-off scenario explored higher costs of treatment for patients with a disease that costs the PBS $5000/saves patients $4960 per month compared with the disease that costs the PBS $100/saves patients $60 per month
Medicines that help patients return to work	Should more PBS money go to patients whose diseases affect their ability to work (Pop 1) compared to those who are able to continue working despite their disease (Pop 2)?	Trade-off scenario explored smaller health gain for disease that impacts upon patients’ ability to work compared with disease that does not prevent patients from working without treatment	Trade-off scenario explored higher costs of treatment for disease that impacts upon patients’ ability to work compared with disease that does not prevent patients from working without treatment
Life style related diseases and individual responsibility	Should more PBS money go to patients with a disease unrelated to lifestyle (Pop 1) compared to those with diseases that are related to lifestyle (Pop 2)?	Trade-off scenario explored smaller health gain for disease that is unrelated to lifestyle compared with the disease that is lifestyle related	Trade-off scenario explored higher costs of treatment for disease that is unrelated to lifestyle compared with the disease that is lifestyle related

^**1**^ Criteria that were the same as those explored in the UK study by Linley et al.

**Abbreviation:** Pop = population

The potential importance of each criterion was quantified by asking each respondent to allocate notional PBS money to combinations of 100 patients, those combinations representing more or fewer patients with a particular criterion (such as patients with severe vs. moderate disease). This was done for all 12 scenarios. For example, if the respondent allocated the PBS budget to 50 patients with moderate disease and 50 patients with severe disease (all else being equal), this indicates indifference to disease importance in the distribution of beneficiaries when allocating the PBS budget. An allocation of more than 50% to patients with severe disease would indicate a societal preference for distributing the drug budget to patients receiving treatment for severe disease i.e. societal preferences were inferred from absolute majority responses for a particular allocation criterion.

The second part of study involved splitting the total respondent sample into two cohorts. Cohort 1 respondents were asked to complete an additional set of trade-offs where the estimate of benefit was varied for each of the two hypothetical patient groups (see S1 File). For cohort 2 respondents, the trade-offs varied according to the cost implications of each criterion (see S2 File). In this way the survey design was consistent with the Linley study [17] and minimised the burden on survey respondents.

**Fig 1:**
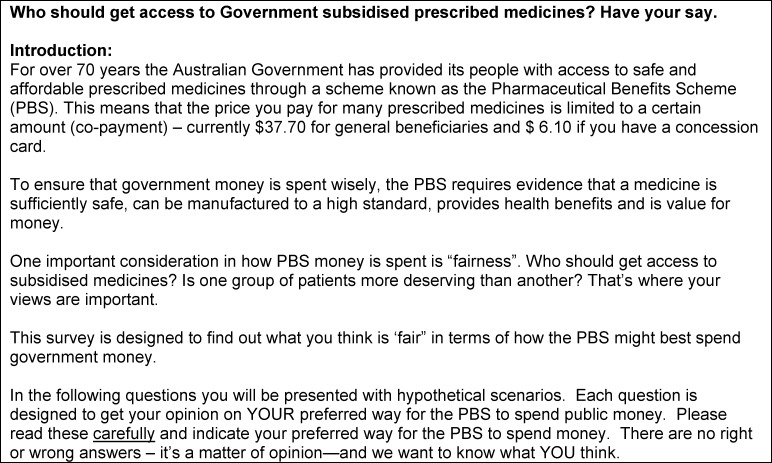
Text introducing the 12 allocation criteria.

**Fig 2:**
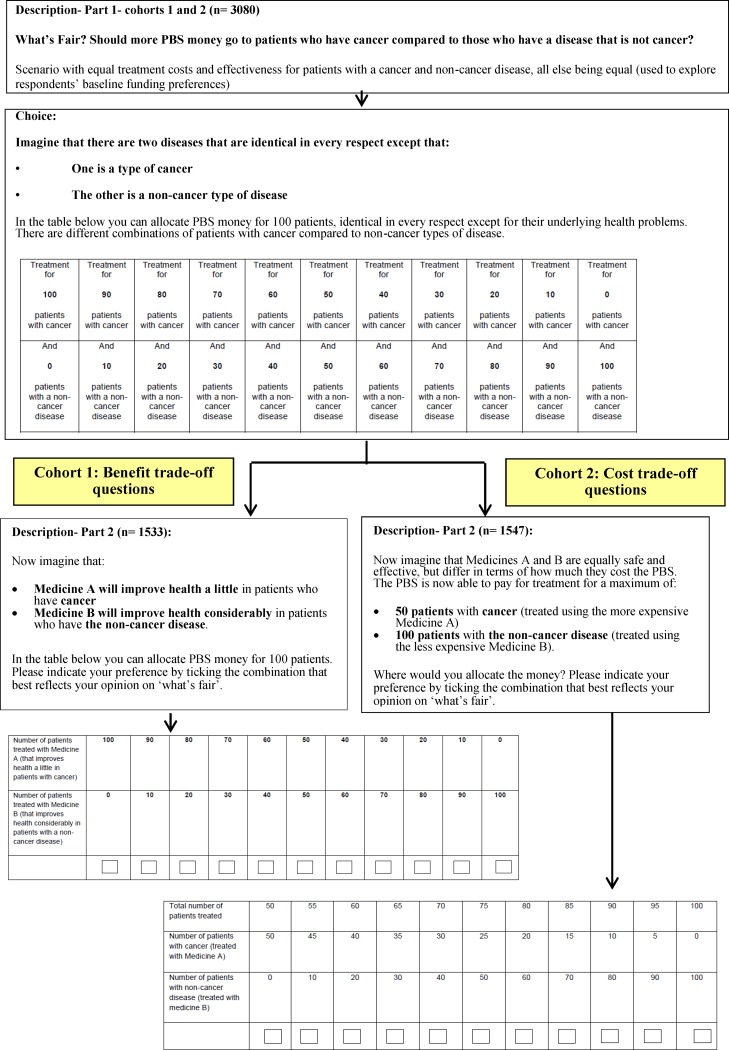
Summary of the survey format using cancer as an example criterion. Socio-demographic data were collected to assess associations between respondents’ characteristics and views on the allocation criterion (see **Table 2**).

**Fig 1** presents the text introducing the 12 allocation criteria. **Fig 2** provides an example of the text of the prioritisation question using cancer treatments as the allocation criterion of interest.

### Administration

#### Participants and recruitment

The target sample size of this study was informed by studies reported in the literature [17, 23, 24] and available resources. The sample of 3080 participants (aged 18 years or older) was drawn from members of the Australian public enrolled on the panel of a market research company. A ‘minimum quota’ approach controlled by gender, age and geographical area (state of residence) was used to ensure that the sample was representative of the general adult Australian population. As described above, participants were divided into two cohorts exploring two different kinds of ‘trade-offs’.

#### Pilot survey

In August 2015, a pilot survey was conducted with 111 participants to test the logistics, flow and user friendliness of the survey. An additional question regarding the state of residence was added after pilot testing. Following completion of pilot testing, the full survey was administered during October 2015 and closed when our target of 3000 complete responses (i.e. 1500 per cohort) was achieved (by the end of the month).

#### Ethics

Ethical approval of the study was obtained from the ethics committee at Sydney University (protocol number: 2014/906).

### Statistical analysis

Descriptive statistics were used to summarize demographic variables. Responses to both parts of the survey (i.e. both the ‘all else being equal’ condition and the trade-off condition) were analyzed by classifying responses into three groups: (1) respondents favoring Population 1; (2) respondents favoring an equal allocation between the two competing populations; (3) respondents favoring Population 2. Societal preferences were inferred from absolute majority responses i.e. populations with more than 50% of respondents’ allocation for a particular allocation criterion. This was repeated for each of the 12 allocation criteria explored. Responses to Part 1 questions from cohorts 1 and 2 were pooled (as both cohorts were asked the same set of ‘all else being equal’ questions). Part 2 results (trade-off questions) were analyzed by cohort. Shift in preferences was determined using each cohort’s preferences under the assumption of ‘all else being equal’ as a baseline. McNemar’s test was used to determine the statistical significance of any relative shifts in preferences between Parts 1 and 2 by cohort. Exact conditional logistic regression was used to obtain odd ratios and 95% confidence intervals.

Logistic regression modeling using gender, age, marital status, education status, health status, cancer history, country of birth, private health status, employment status, household income, dependent children and state of residence was conducted to determine their impact on respondents’ expressed baseline funding preferences on the 12 allocation criteria (i.e. under the assumption of ‘all else being equal’). Model fit was tested using the Hosmer and Lemeshow [25] goodness-of-fit test. All statistical analyses ware performed using version 9.4 of SAS.

## Results

### Demographics

A total of 3080 adult members of the general public in Australia completed the on-line survey. The second part of the survey–the benefit and cost trade-off scenarios–required splitting the sample into two equal sized cohorts. The characteristics of the respondents in each of the cohorts were almost identical (**Table 2**).

**Table 2:** Characteristics of respondents (N = 3080).

	Cohort 1		Cohort 2		Combined		Australia^3^
Characteristics	(N = 1533)		(N = 1547)		(N = 3080)		
	n	%	n	%	n	%	%
**Gender**							
Male	749	48.9	753	48.7	1502	48.8	48.9
Female	784	51.1	794	51.3	1578	51.2	51.1
**Age (years)**							
18–24	186	12.1	188	12.2	374	12.1	12.2
25–34	268	17.5	274	17.7	542	17.6	18.0
35–44	299	19.5	297	19.2	596	19.4	18.5
45–54	276	18.0	277	17.9	553	18.0	17.9
55–64	240	15.7	241	15.6	481	15.6	15.2
65+	264	17.2	270	17.5	534	17.3	18.2
**Marital status**							
Married/de facto	908	59.2	924	59.7	1832	59.5	
Separated/divorced	156	10.2	152	9.8	308	10.0	
Widowed	55	3.6	43	2.8	98	3.2	
Never married	414	27.0	428	27.7	842	27.3	
**Education**							
Never attended school/ primary/ some high school	211	13.8	220	14.2	431	14.0	
Completed high school	318	20.7	309	20.0	627	20.4	
University, TAFE etc.	998	65.1	1011	65.4	2009	65.2	
Prefer not to answer	6	0.4	7	0.5	13	0.4	
**Cancer history**							
Cancer history with death^1^	597	38.9	578	37.4	1175	38.1	
Cancer history with no death/death unknown	243	15.9	246	15.9	489	15.9	
No cancer history	673	43.9	703	45.4	1376	44.7	
Prefer not to answer	20	1.3	20	1.3	40	1.3	
**General health**							
Very good	267	17.4	277	17.9	544	17.7	
Good	750	48.9	731	47.3	1481	48.1	
Average	408	26.6	434	28.1	842	27.3	
Poor/ very poor	108	7.0	105	6.8	213	6.9	
**Country of birth**							
Australia	1141	74.4	1144	73.9	2285	74.2	
Overseas	392	25.6	403	26.1	795	25.8	
**Private health insurance**							
Yes	896	58	918	59	1814	59	
No	637	42	629	41	1266	41	
**Employment status**							
Working full time	546	35.6	536	34.6	1082	35.1	
Working part time	303	19.8	319	20.6	622	20.2	
Currently not working, but looking for work	179	11.7	197	12.7	376	12.2	
Retired	327	21.3	342	22.1	669	21.7	
Other	178	11.6	153	9.9	331	10.7	
**Household annual income**							
$0 to 20,000	120	7.8	129	8.3	249	8.1	
$20,001–40,000	310	20.2	300	19.4	610	19.8	
$40,001 to 80,000	427	27.9	436	28.2	863	28.0	
$80,001 to 180,000	436	28.4	438	28.3	874	28.4	
$180,001 and over	65	4.2	69	4.5	134	4.4	
Prefer not to answer	175	11.4	175	11.3	350	11.4	
**Personal annual income**							
$0 to 20,000	380	24.8	374	24.2	754	24.5	
$20,001–40,000	364	23.7	347	22.4	711	23.1	
$40,001 to 80,000	395	25.8	397	25.7	792	25.7	
$80,001 to 180,000	203	13.2	219	14.2	422	13.7	
$180,001 and over	23	1.5	24	1.6	47	1.5	
Prefer not to answer	168	11.0	186	12.0	354	11.5	
**Household composition**							
With financially dependent children	453	29.5	474	30.6	927	30.1	
Without financially dependent children	1080	70.5	1073	69.4	2153	69.9	
**State**							
Australian Capital Territory	24	1.6	23	1.5	47	1.5	1.7
New South Wales	496	32.4	489	31.6	985	32.0	32.2
Northern Territory	3	0.2	7	0.5	10	0.3	0.9
Queensland	292	19.0	295	19.1	587	19.1	19.9
South Australia	117	7.6	119	7.7	236	7.7	7.6
Tasmania	36	2.3	34	2.2	70	2.3	2.3
Victoria	368	24.0	377	24.4	745	24.2	25.1
Western Australia	142	9.3	147	9.5	289	9.4	10.4
Unknown^2^	55	3.6	56	3.6	111	3.6	-

^1^ The variable ‘cancer history with death’ pertains to cancer related deaths in close family members of the survey respondents.

^**2**^ The pilot survey did not include this demographic question (n = 111).

^**3**^ Australia demographics (gender, age and state of residence) are for persons aged 18 years and over, sourced from the TableBuilder available from the Australian Bureau of Statistics based on the 2011 Census data (http://www.abs.gov.au/websitedbs/censushome.nsf/home/tablebuilder?opendocument&navpos=240). TableBuilder is an online self-help tool that enables users to create tables, graphs and maps of Census data.

### All respondents: Allocation preferences under the assumption of ‘all else being equal’

**Table 3** summarises respondents’ baseline (“all else being equal”) preferences for allocating PBS funds between two competing populations according to each of the 12 allocation criteria.

**Table 3:** Respondents’ preferences by scenarios: (1) all else being equal and (2) benefit and cost trade-offs.

Scenario population 1	Cohort	Choice	Prioritise population 1	Equal allocation to both populations	Prioritise population 2	Choice	Scenario population 2
			N (percentage, 95% CI)	N (percentage, 95% CI)	N (percentage, 95% CI)		
**Severe disease**	**Com**	**All else being equal**^**1**^	**1624 (52.7, 51.0–54.5)**	1286 (41.8, 40.0–43.5)	170 (5.5, 4.7–6.4)	**All else being equal**^**1**^	**Moderate disease**
	1	Little health improvement	392 (25.6, 23.4–27.8)	700 (45.7, 43.2–48.2)	441 (28.8, 26.5–31.1)	improves health considerably	
			OR = 0.14; p <0.001	OR = 1.32; p = 0.004	OR = 9.66; p <0.001		
	2	Twice the cost of population 2	751 (48.5, 46.0–51.1)	663 (42.9, 40.4–45.3)	133 (8.6, 7.3–10.0)	Half the cost of population 1	
			OR = 0.70; p<0.001	OR = 1.07; p = 0.52	OR = 2.49; p<0.001		
**No other medicine available**	**Com**	**All else being equal**^**1**^	**1652 (53.6,51.9–55.4)**	1121 (36.4, 34.7–38.1)	307 (10.0, 8.9–11.1)	**All else being equal**^**1**^	**Several other medicines available**
	1	Little health improvement	639 (41.7, 39.2–44.2)	594 (38.7, 36.3–41.2)	300 (19.6, 17.6–21.7)	improves health considerably	
			OR = 0.38; p <0.001	OR = 1.25; p = 0.04	OR = 3.20; p <0.001		
	2	Twice the cost of population 2	867 (56.0, 53.5–58.5)	519 (33.5, 31.2–36.0)	161 (10.4, 8.9–12.0)	Half the cost of population 1	
			OR = 1.26; p = 0.04	OR = 0.73; p = 0.007	OR = 1.10; p = 0.57		
**Medicines work in a new way**	**Com**	**All else being equal** ^**1**^	1213 (39.4, 37.7–41.1)	1523 (49.4, 47.7–51.2)	344 (11.2, 10.1–12.3)	**All else being equal**^**1**^	**Medicines work in a similar way to existing medicines**
	1	Little health improvement	477 (31.1, 28.8–33.5)	599 (39.1, 36.6–41.6)	457 (29.8, 27.5–32.2)	improves health considerably	
			OR = 0.51; p <0.001	OR = 0.50; p <0.001	OR = 5.71; p <0.001		
	2	Twice the cost of population 2	675 (43.6, 41.1–46.2)	583 (37.7, 35.3–40.2)	289 (18.7, 16.8–20.7)	Half the cost of population 1	
			OR = 1.55; p < 0.001	OR = 0.42; p < 0.001	OR = 2.19; p <0.001		
**Patients reliant on carers for their day-to-day needs**	**Com**	**All else being equal**^**1**^	1204 (39.1, 37.4–40.8)	1342 (43.6, 41.8–45.3)	534 (17.3, 16.0–18.7)	**All else being equal**^**1**^	**Patients remain independent**
	1	Little health improvement	483 (31.5, 29.2–33.9)	584 (38.1, 35.7–40.6)	466 (30.4, 28.1–32.8)	improves health considerably	
			OR = 0.45;p <0.001	OR = 0.72; p = 0.003	OR = 3.19; p <0.001		
	2	Twice the cost of population 2	673 (43.5, 41.0–46.0)	591 (38.2, 35.8–40.7)	283 (18.3, 16.4–20.3)	Half the cost of population 1	
			OR = 1.72; p < 0.001	OR = 0.53; p <0.001	OR = 1.21; p = 0.19		
**Patients who are not financially well off**	**Com**	**All else being equal**^**1**^	**1920 (62.3, 60.6–64.1)**	931 (30.2, 28.6–31.9)	229 (7.4, 6.5–8.4)	**All else being equal**^**1**^	**Patients who are financially well off**
	1	Little health improvement	801 (52.3, 49.7–54.8)	558 (36.4); 34.0–38.9)	174 (11.4, 9.8–13.1)	improves health considerably	
			OR = 0.35; p <0.001	OR = 2.02; p <0.001	OR = 2.07; p <0.001		
	2	Twice the cost of population 2	995 (64.3, 61.9–66.7)	420 (27.1, 25.0–29.4)	132 (8.5, 7.2–10.0	Half the cost of population 1	
			OR = 1.30; p = 0.03	OR = 0.67; p = 0.001	)OR = 1.22; p = 0.28		
**Children**	**Com**	**All else being equal**^**1**^	1171 (38.0, 36.3–39.8)	1696 (55.1, 53.5–56.8)	213 (6.9, 6.0–7.9)	**All else being equal**^**1**^	**Adults**
	1	Little health improvement	440 (28.7, 26.5–31.0)	748 (48.8, 46.3–51.3)	345 (22.5, 20.4–24.7)	improves health considerably	
			OR = 0.44; p < 0.001	OR = 0.59; p <0.001	OR = 6.93; p <0.001		
	2	Twice the cost of population 2	808 (52.2, 49.7–54.8)	624 (40.3, 37.9–42.8)	115 (7.4, 6.2–8.9)	Half the cost of population 1	
			OR = 3.45; p <0.001	OR = 0.29; p <0.001	OR = 0.97; p = 0.93		
**18 months survival without treatment (End of life)**	**Com**	**All else being equal**^**1**^	814 (26.4, 24.9–28.0)	1532 (49.7, 48.0–51.5)	734 (23.8, 22.3–25.4)	**All else being equal**^**1**^	**60 months survival without treatment**
	1	3 month survival gain	371 (24.2, 22.1–26.4)	839 (54.7, 52.2–57.2)	323 (21.1, 19.1–23.2)	6 month survival gain	
			OR = 1.00; p = 1.00	OR = 1.31; p = 0.01	OR = 0.68; p = 0.003		
	2	Twice the cost of population 2	604 (39.0, 36.6–41.5)	649 (42.0, 39.5–44.5)	294 (19.0, 17.1–21.1	Half the cost of population 1	
			OR = 3.73; p <0.001	OR = 0.57; P < 0.001	)OR = 0.52; P <0.001		
**Cancer**	**Com**	**All else being equal**^**1**^	1049 (34.1, 32.4–35.8)	1773 (57.6, 55.8–59.3)	258 (8.4, 7.4–9.4)	**All else being equal**^**1**^	**Non-cancer disease**
	1	Little health improvement	426 (27.8, 25.6–30.1)	697 (45.5, 43.0–48.0)	410 (26.7, 24.5–29.0)	improves health considerably	
			OR = 0.58; p <0.001	OR = 0.37; p <0.001	OR = 8.05; p <0.001		
	2	Twice the cost of population 2	731 (47.3, 44.7–49.8)	651 (42.1, 39.6–44.6)	165 (10.7, 9.2–12.3)	Half the cost of population 1	
			OR = 3.02; p <0.001	OR = 0.32; p <0.001	OR = 1.21; p = 0.29		
**Rare disease**	**Com**	**All else being equal**^**1**^	800 (26.0, 24.4–27.6)	1311 (42.6, 40.8–44.3)	969 (31.5, 29.8–33.1)	**All else being equal**^**1**^	**Common disease**
	1	Little health improvement	345 (22.5, 20.4–24.7)	574 (37.4, 35.0–39.9)	614 (40.1, 37.6–42.6)	improves health considerably	
			OR = 0.67; p = 0.003	OR = 0.74; p = 0.01	OR = 2.10; p <0.001		
	2	Twice the cost of population 2	564 (36.5, 34.1–38.9)	603 (39.0, 36.5–41.5)	380 (24.6, 22.4–26.8)	Half the cost of population 1	
			OR = 3.14; p <0.001	OR = 0.59; p <0.001	OR = 0.54; p <0.001		
**costs the PBS $5000/saves patients $4960 per month**	**Com**	**All else being equal**^**1**^	1264 (41.0, 39.3–42.8)	1357 (44.1, 42.3–45.8)	459 (14.9, 13.7–16.2)	**All else being equal**^**1**^	**costs the PBS $100 per month to subsidise and saves patients $60**
	1	Little health improvement	478 (31.2, 28.9–33.6)OR = 0.34; p <0.001	578 (37.7, 35.3–40.2)OR = 0.70; p = 0.001	477 (31.1, 28.8–33.5)OR = 5.00; p <0.001	improves health considerably	
	2	***No cost trade-off question for cohort 2***					
**Patients unable to work without treatment**	**Com**	**All else being equal**^**1**^	1441 (46.8, 45.0–48.6)	1225 (39.8, 38.0–41.5)	414 (13.4, 12.3–14.7)	**All else being equal**^**1**^	**Patients able to work without treatment**
	1	Little health improvement	566 (36.9, 34.5–39.4)	643 (41.9, 39.5–44.5)	324 (21.1, 19.1–23.3)	improves health considerably	
			OR = 0.42; p <0.001	OR = 1.39; p = 0.002	OR = 2.10; p <0.001		
	2	Twice the cost of population 2	779 (50.4, 47.8–52.9)	569 (36.8, 34.4–39.2)	199 (12.9, 11.2–14.6)	Half the cost of population 1	
			OR = 1.54; p<0.001	OR = 0.63; p <0.001	OR = 1.06; p = 0.77		
**Patients whose disease is unrelated to life-style**	**Com**	**All else being equal**^**1**^	**1593 (51.7, 49.9–53.5)**	1189 (38.6, 36.9–40.4)	296 (9.7, 8.7–10.8)	**All else being equal**^**1**^	**Patients whose disease is related to life-style**
	1	Little health improvement	650 (42.4, 39.9–44.9)	641 (41.8, 39.3–44.3)	242 (15.8, 14.0–17.7)	improves health considerably	
			OR = 0.40; p <0.001	OR = 1.34; p = 0.01	OR = 2.51; p <0.001		
	2	Twice the cost of population 2	899 (58.1, 55.6–60.6)	502 (32.4, 30.1–34.9)	146 (9.4, 8.0–11.0)	Half the cost of population 1	
			OR = 1.94; p <0.001	OR = 0.51; p <0.001	OR = 0.93; p = 0.74		

^1^ Pooled results of cohorts 1 and 2 (n = 3080).

**Abbreviation:** Com = combined cohorts 1 and 2

### Allocation criteria considered more important than their alternatives (i.e. with more than 50% of respondents’ allocation)

Of the allocation criteria explored, all else being equal, respondents expressed a preference (inferred from absolute majority responses) for allocating PBS money on medicines (1) treating severe diseases (as opposed to moderate diseases): 52.7%, (2) treating diseases for which there is no alternative treatment available on the PBS (compared to those where several alternative treatments are available): 53.6%, (3) treating diseases that affect patients who are not financially well off (as opposed to those that affect patients who are financially well off): 62.3%, and (4) treating life-style unrelated diseases (rather than life-style related diseases): 51.7%.

#### Allocation criteria considered equally important

All else being equal, between 55.1 to 57.6% of respondents divided resources evenly on medicines treating: (1) diseases affecting children vs. adults (55.1%) and (2) cancer vs. non-cancer (57.6%).

#### Benefit and cost trade-off scenarios

**Table 3** summarises the effects of varying health gains (Cohort 1) and treatment costs (Cohort 2) on respondents’ allocation preferences for each of the 12 allocation criteria explored.

#### Effect of varying health gains on respondents’ allocation preferences (benefit trade-off)

A total of 1533 respondents (Cohort 1) completed the benefit trade-off scenarios for the 12 allocation criteria explored. This group was asked to reassess their original allocations on the assumption that one population would gain a small health improvement, while the other would gain a large health improvement.

Removing the assumption of equal treatment effectiveness resulted in a statistically significant shift in respondents’ allocation preferences away from the population that gained a ‘little health improvement’ towards the population that gained a ‘considerable health improvement’ for all criteria with the exception of ‘end-of-life treatments’. Results for the ‘end-of-life treatments’ criterion indicated a shift in respondents’ preferences away from the ‘considerable health improvement’ population to favouring an equal allocation between the two competing populations under the benefit trade-off condition. However, the proportion of respondents favouring the population that gained a ‘little health improvement’ remained unchanged when compared to the ‘all else being equal’ assumption (24.2% vs. 24.2%, OR = 1.00, p = 1.00).

Whilst there was an overall shift away from the ‘little’ to ‘considerable’ health improvement population, between 42.4 to 52.3% of respondents remained in favour of treating the former. This was despite the assumption that these patients would derive a little improvement in health compared with a considerable health improvement for the following allocation criteria: (1) treating diseases for which there is no alternative treatment available on the PBS instead of diseases for which several alternative treatments are available (47.1%), (2) treating diseases that affect patients who are not financially well off rather than the financially well off (52.3%), and (3) treating life-style unrelated diseases rather than the life-style related diseases (42.4%).

#### Effect of varying treatment costs on respondents’ allocation preferences (cost trade-off)

A total of 1547 respondents (Cohort 2) completed the cost trade-off scenarios for the 12 allocation criteria explored. This group was asked to reassess their original allocations on the assumption that one population would be more costly to treat than the other. Therefore, the cost trade-off scenarios represent a trade-off in the total number of patients who could be treated.

Responses to the cost trade-off scenarios (n = 1547) indicated a significant reduction in the proportion of respondents choosing to divide resources equally and a shift in preference towards allocating resources to the populations that were more costly to treat for all allocation criteria with the exception of severity of disease. Despite the increased treatment costs and the resulting decreased number of total patients who can be treated with the available resources, 50% or more of the respondents expressed a preference for allocating greater amounts of PBS money on medicines (1) treating diseases for which there is no alternative treatment available on the PBS instead of diseases where several alternative treatments are available (56.0%), (2) treating diseases that affect patients who are not financially well off rather than those that affect patients who are financially well off (64.3%), (3) treating children instead of adult patients (52.2%), (4) treating patients whose diseases affect their ability to work as opposed to those who are able to work (50.4%), and (5) treating life-style unrelated diseases rather than diseases that are related to life-style related diseases (58.1%).

### Relationship between respondents characteristics and allocation preferences

Multivariable logistic regression for each of the 12 allocation criteria was conducted in order to investigate if there was a difference between allocation preferences (favouring population 1 versus equal allocation versus favouring population 2) under the assumption of ‘all else being equal’, after adjusting for confounders. Results suggested that respondents’ preferences for allocation were influenced by their individual characteristics and circumstances. The results are summarised in **Table 4**.

**Table 4:** Multivariate Analysis under assumption of equal treatment effectiveness and costs.

Explanatory variables	Dependent variables favoured versus (equal and not favoured)											
	Severity of disease, ORs (95% CIs)	No alternate medicine, ORs (95% CIs)	Innovative medicineORs (95% CIs)	Carer burden, ORs (95% CIs)	Not financially well off, ORs (95% CIs)	Children, ORs (95% CIs)	End of life therapies, ORs (95% CIs)	Cancer diseases, ORs (95% CIs)	Rare diseases, ORs (95% CIs)	Cost to the PBS, ORs (95% CIs)	Productivity- patient unable to work without treatment, ORs (95% CIs)	Life style unrelated diseases, ORs (95% CIs)
**Gender**												
Male	1 (referent)	1 (referent)	1 (referent)	1 (referent)	1 (referent)	1 (referent)	1 (referent)	1 (referent)	1 (referent)	1 (referent)	1 (referent)	1 (referent)
	**p = 0.0095**	**p = 0.011**	p = 0.25	p = 0.16	p = 0.32	**p = 0.0002**	p = 0.02	p = 0.49	p = 0.85	p = 0.30	p = 0.64	p = 0.88
Female	0.82	1.22	0.95	0.91	1.08	0.74	0.81	0.95)	0.98	0.92	1.04	0.99
	(0.7–0.95)	(1.05–1.42)	(0.81–1.11)	(0.78–1.07)	(0.93–1.27)	(0.63–0.86)	(0.68–0.97)	(0.81–1.11	(0.83–1.17)	(0.79–1.08)	(0.89–1.21)	(0.85–1.15)
**Age**												
18–24	1 (referent)	1 (referent)	1 (referent)	1 (referent)	1 (referent)	1 (referent)	1 (referent)	1 (referent)	1 (referent)	1 (referent)	1 (referent)	1 (referent)
	**p = 0.007**	p = 0.92	**p = 0.04**	**p = 0.04**	**p = 0.009**	**p = 0.001**	**p<0.0001**	p = 0.32	**p = 0.02**	p = 0.71	p = 0.66	p = 0.30
25–34	0.78	0.92	0.96	1	0.89	0.73	0.76	1.08	0.77	0.95	0.91	0.98
	(0.58–1.05)	(0.68–1.23)	(0.71–1.29)	(0.74–1.34)	(0.66–1.2)	(0.54–0.99)	(0.56–1.03)	(0.8–1.47)	(0.56–1.06)	(0.71–1.27)	(0.68–1.22)	(0.73–1.32)
35–44	0.8	0.86	0.88	0.7	1.06	0.61	0.57	0.88	0.59	0.83	0.93	1.02
	(0.59–1.08)	(0.64–1.16)	(0.65–1.19)	(0.52–0.95)	(0.78–1.44)	(0.45–0.83)	(0.42–0.78)	(0.64–1.19)	(0.42–0.82)	(0.61–1.12)	(0.69–1.25)	(0.76–1.36)
45–54	0.64	0.9	0.68	0.76	0.97	0.51	0.5	0.86	0.67	0.86	0.99	0.93
	(0.47–0.87)	(0.67–1.22)	(0.5–0.93)	(0.56–1.03)	(0.71–1.32)	(0.37–0.7)	(0.36–0.69)	(0.62–1.18)	(0.48–0.94)	(0.64–1.17)	(0.73–1.33)	(0.69–1.26)
55–64	0.56	0.95	0.94	0.72	1.1	0.64	0.34	0.9	0.62	0.84	0.81	0.91
	(0.4–0.79)	(0.68–1.32)	(0.67–1.32)	(0.51–1.01)	(0.78–1.55)	(0.46–0.91)	(0.23–0.5)	(0.64–1.28)	(0.43–0.91)	(0.6–1.17)	(0.58–1.12)	(0.66–1.27)
65+	0.74	1.02	0.88	0.72	1.78	0.76	0.41	1.1	0.8	0.95	0.96	1.28
	(0.5–1.1)	(0.69–1.51)	(0.59–1.3)	(0.49–1.08)	(1.18–2.7)	(0.5–1.13)	(0.26–0.64)	(0.73–1.66)	(0.51–1.25)	(0.64–1.42)	(0.65–1.42)	(0.86–1.9)
**Marital status**												
Married /de facto	1 (referent)	1 (referent)	1 (referent)	1 (referent)	1 (referent)	1 (referent)	1 (referent)	1 (referent)	1 (referent)	1 (referent)	1 (referent)	1 (referent)
	p = 0.11	p = 0.42	p = 0.93	p = 0.88	p = 0.43	p = 0.03	p = 0.18	p = 0.65	p = 0.14	p = 0.31	p = 0.77	p = 0.28
Separated/divorced	1.27	1.05	1.12	1.05	1.26	1.17	1.31	0.96	0.72	1.09	1.05	1.27
	(0.98–1.62)	(0.81–1.36)	(0.86–1.45)	(0.8–1.36)	(0.96–1.65)	(0.9–1.52)	(0.98–1.76)	(0.73–1.25)	(0.53–0.99)	(0.84–1.41)	(0.81–1.36)	(0.98–1.64)
Widowed	0.74	1.15	0.95	1.01	0.98	1.54	1.12	1.01	0.75	1.51	0.82	0.92
	(0.48–1.21)	(0.74–1.78)	(0.61–1.48)	(0.64–1.58)	(0.62–1.56)	(0.99–2.38)	(0.67–1.9)	(0.64–1.58)	(0.45–1.26)	(0.98–2.32)	(0.53–1.27)	(0.6–1.43)
Never married	1.10	1.19	0.94	0.95	1.06	0.82	0.91	0.86	0.86	1.03	1.03	1.00
	(0.89–1.39)	(0.96–1.47)	(0.76–1.17)	(0.76–1.18)	(0.85–1.32)	(0.65–1.03)	(0.71–1.16)	(0.71–1.16)	(0.68–1.1)	(0.83–1.28)	(0.84–1.27)	(0.81–1.23)
**Education**												
Never attended/ primary or some high school	1 (referent)	1 (referent)	1 (referent)	1 (referent)	1 (referent)	1 (referent)	1 (referent)	1 (referent)	1 (referent)	1 (referent)	1 (referent)	1 (referent)
	p = 0.64	p = 0.39	p = 0.10	p = 0.24	p = 0.30	p = 0.18	p = 0.19	p = 0.04	p = 0.06	p = 0.16	p = 0.53	P = 0.29
Completed high school	0.90	1.22	1.00	1.11	1.26	0.81	1.19	1.07	0.92	1.22	1.03	1.12
	(0.7–1.16)	(0.95–1.58)	(0.77–1.3)	(0.86–1.44)	(0.97–1.65)	(0.62–1.05)	(0.89–1.59)	(0.82–1.39)	(0.69–1.22)	(0.94–1.59)	(0.8–1.34)	(0.86–1.44)
Uni/Tafe	0.87	1.2	0.95	0.94	1.14	0.84	0.94	0.82	0.75	1.13	1	1.23
	(0.69–1.08)	(0.96–1.5)	(0.76–1.2)	(0.74–1.18)	(0.9–1.43)	(0.67–1.06)	(0.73–1.23)	(0.65–1.03)	(0.59–0.97)	(0.9–1.42)	(0.8–1.25)	(0.99–1.54)
Preferred not to answer	0.77	0.98	0.73	0.24	0.69	0.27	0.78	0.73	0.49	0.3	0.37	1.29
	(0.24–2.5)	(0.3–3.15)	(0.21–2.52)	(0.05–1.17)	(0.21–2.27)	(0.06–1.28)	(0.2–3.09)	(0.21–2.57)	(0.12–1.99)	(0.06–1.44)	(0.1–1.45)	(0.4–4.18)
**General health (self-reported)**												
Very good	1 (referent)	1 (referent)	1 (referent)	1 (referent)	1 (referent)	1 (referent)	1 (referent)	1 (referent)	1 (referent)	1 (referent)	1 (referent)	1 (referent)
	p = 0.12	p = 0.66	p = 0.33	p = 0.31	p = 0.22	p = 0.36	p = 0.41	p = 0.63	p = 0.27	p = 0.91	p = 0.21	p = 0.38
Good	1.19	1.13	1.25	1.15	1.2	1.18	0.94	1.14	1.2	1.04	1.24	1.01
	(0.98–1.46)	(0.92–1.38)	(1.02–1.54)	(0.93–1.41)	(0.97–1.47)	(0.96–1.46)	(0.75–1.18)	(0.92–1.41)	(0.95–1.51)	(0.85–1.28)	(1.01–1.51)	(0.82–1.23)
Average	1.07	1.06	1.23	1.17	1.1	1.12	0.95	1.09	1.01	1.09	1.21	0.87
	(0.85–1.34)	(0.84–1.32)	(0.98–1.55)	(0.93–1.47)	(0.87–1.39)	(0.89–1.42)	(0.74–1.23)	(0.86–1.38)	(0.78–1.32)	(0.86–1.36)	(0.97–1.52)	(0.69–1.08)
Poor/very poor	1.40	1.11	0.96	0.94	1.35	0.99	1.24	1.17	1.13	1.03	1.24	0.95
	(1–1.96)	(0.79–1.54)	(0.68–1.36)	(0.67–1.33)	(0.95–1.93)	(0.70–1.41)	(0.86–1.79)	(0.83–1.66)	(0.77–1.66)	(0.73–1.44)	(0.89–1.74)	(0.68–1.33)
**Cancer History**												
Cancer history with death ^**1**^	1 (referent)	1 (referent)	1 (referent)	1 (referent)	1 (referent)	1 (referent)	1 (referent)	1 (referent)	1 (referent)	1 (referent)	1 (referent)	1 (referent)
	p = 0.71	p = 0.48	p = 0.89	p = 0.80	**p = 0.02**	p = 0.62	p = 0.85	p = 0.24	p = 0.15	p = 0.40	p = 0.18	p = 0.68
Cancer history- no death or death unknown	0.89	1.04	0.86	0.96	0.90	0.99	0.98	0.91	0.83	0.86	1.02	1.02
	(0.72–1.11)	(0.84–1.29)	(0.69–1.07)	(0.77–1.2)	(0.72–1.12)	(0.79–1.24)	(0.77–1.25)	(0.72–1.13)	(0.65–1.07)	(0.69–1.07)	(0.82–1.26)	(0.82–1.27)
No cancer history	0.93	0.91	0.91	1.04	0.77	1.06	0.92	0.84	0.97	0.9	0.86	0.93
	(0.79–1.09)	(0.77–1.06)	(0.77–1.07)	(0.88–1.23)	(0.65–0.90)	(0.90–1.25)	(0.77–1.11)	(0.71–1.00)	(0.81–1.16)	(0.76–1.06)	(0.73–1.01)	(0.79–1.09)
Preferred not to answer	0.98	1.10	0.93	1.07	0.70	0.69	0.87	1.08	1.78	1.17	1.24	0.78
	(0.50–1.93)	(0.56–2.17)	(0.47–1.85)	(0.54–2.12)	(0.35–1.37)	(0.33–1.42)	(0.41–1.82)	(0.54–2.16)	(0.89–3.54)	(0.59–2.32)	(0.63–2.44)	(0.40–1.53)
**Country of birth**												
Australia	1 (referent)	1 (referent)	1 (referent)	1 (referent)	1 (referent)	1 (referent)	1 (referent)	1 (referent)	1 (referent)	1 (referent)	1 (referent)	1 (referent)
	p = 0.76	p = 0.86	p = 0.74	p = 0.63	p = 0.88	p = 0.12	p = 0.42	p = 0.30	p = 0.41	p = 0.63	p = 0.11	p = 0.19
overseas	1.03	0.99	1.25	1.03	1.01	1.15	0.92	1.10	1.08	0.96	0.87	0.89
	(0.87–1.22)	(0.83–1.17)	(1.05–1.48)	(0.87–1.22)	(0.85–1.21)	(0.96–1.36)	(0.76–1.12)	(0.92–1.31)	(0.9–1.31)	(0.81–1.14)	(0.74–1.03)	(0.75–1.06)
**Health insurance**												
Yes	1 (referent)	1 (referent)	1 (referent)	1 (referent)	1 (referent)	1 (referent)	1 (referent)	1 (referent)	1 (referent)	1 (referent)	1 (referent)	1 (referent)
	p = 0.37	p = 0.71	p = 0.32	p = 0.31	p = 0.39	p = 0.27	p = 0.15	p = 0.95	p = 0.68	p = 0.20	**p = 0.002**	**p = 0.0002**
No	0.93	0.97	1.01	0.92	1.07	1.1	0.88	1.01	1.04	0.9	0.77	0.74
	(0.79–1.09)	(0.83–1.14)	(0.86–1.18)	(0.78–1.08)	(0.91–1.27)	(0.93–1.29)	(0.73–1.05)	(0.85–1.19)	(0.87–1.24)	(0.77–1.06)	(0.66–0.91)	(0.64–0.87)
**Employment status**												
Full time	1 (referent)	1 (referent)	1 (referent)	1 (referent)	1 (referent)	1 (referent)	1 (referent)	1 (referent)	1 (referent)	1 (referent)	1 (referent)	1 (referent)
	p = 0.07	p = 0.10	p = 0.29	p = 0.4990	**p = 0.0002**	**p = 0.04**	**p = 0.02**	p = 0.48	p = 0.61	**p = 0.013**	p = 0.03	**p< 0.0001**
Part time	1.32	1.1	1.32	1.07	1.27	1.39	1.13	1.02	0.86	1.25	1.07	1.54
	(1.06–1.64)	(0.88–1.36)	(1.06–1.65)	(0.86–1.34)	(1.02–1.59)	(1.11–1.74)	(0.89–1.45)	(0.81–1.28)	(0.67–1.11)	(1.00–1.55)	(0.86–1.33)	(1.24–1.92)
Not working	1.34	1.09	1.09	1.19	1.42	1.29	1.64	1	1.02	1.19	1.29	1.24
	(1.03–1.76)	(0.83–1.42)	(0.83–1.43)	(0.9–1.55)	(1.08–1.87)	(0.98–1.7)	(1.23–2.19)	(0.75–1.32)	(0.76–1.38)	(0.91–1.56)	(0.99–1.69)	(0.95–1.62)
Retired	1.05	1.28	1.16	0.94	1.02	1.39	1.06	0.79	0.81	1.03	1.06	1.11
	(0.77–1.42)	(0.95–1.74)	(0.85–1.58)	(0.69–1.28)	(0.74–1.39)	(1.01–1.9)	(0.73–1.53)	(0.57–1.09)	(0.57–1.15)	(0.76–1.41)	(0.78–1.44)	(0.82–1.51)
Other	1.16	1.45	1.41	1.28	1.9	1.39	1.25	1.06	0.91	1.58	1.53	1.87
	(0.88–1.53)	(1.10–1.92)	(1.07–1.87)	(0.97–1.7)	(1.41–2.56)	(1.04–1.85)	(0.92–1.71)	(0.80–1.41)	(0.67–1.24)	(1.20–2.08)	(1.16–2.01)	(1.42–2.48)
**Household income**												
$0 to 20,000	1 (referent)	1 (referent)	1 (referent)	1 (referent)	1 (referent)	1 (referent)	1 (referent)	1 (referent)	1 (referent)	1 (referent)	1 (referent)	1 (referent)
	**p<0.0001**	**p<0.0001**	p = 0.21	p = 0.15	p = 0.28	**p = 0.008**	p = 0.35	p = 0.81	p = 0.58	p = 0.0721	p = 0.18	p = 0.25
$20,001 to 40,000	1.43	1.08	1.29	1.37	1.35	0.89	0.92	0.86	0.87	0.98	0.98	1.14
	(1.05–1.95)	(0.8–1.47)	(0.94–1.77)	(1–1.89)	(0.98–1.87)	(0.65–1.22)	(0.65–1.29)	(0.62–1.18)	(0.61–1.22)	(0.72–1.33)	(0.72–1.34)	(0.84–1.55)
$40,001 to 80,000	1.47	1.24	1.2	1.19	1.26	1.01	0.89	0.92	0.86	1.12	1.06	1.22
	(1.08–2)	(0.92–1.68)	(0.88–1.65)	(0.86–1.63)	(0.92–1.73)	(0.73–1.38)	(0.64–1.25)	(0.67–1.26)	(0.61–1.21)	(0.82–1.52)	(0.78–1.44)	(0.9–1.66)
$80,001 to 180,000	2.12	1.64	1.43	1.40	1.33	1.42	0.95	0.92	0.81	1.23	1.14	1.42
	(1.52–2.94)	(1.19–2.28)	(1.02–2.00)	(1–1.96)	(0.95–1.86)	(1.02–1.99)	(0.66–1.36)	(0.65–1.28)	(0.56–1.16)	(0.88–1.71)	(0.82–1.58)	(1.03–1.97)
$180,001 and over	2.50	2.14	1.69	1.57	1.37	1.15	1.02	0.80	0.85	1.31	1.04	1.57
	(1.56–4)	(1.34–3.42)	(1.06–2.7)	(0.98–2.51)	(0.85–2.2)	(0.71–1.84)	(0.62–1.7)	(0.5–1.31)	(0.51–1.43)	(0.83–2.08)	(0.66–1.65)	(0.99–2.49)
Preferred not to answer	1.13	0.94	1.05	1.29	1.06	1.07	0.69	0.80	0.69	0.82	0.79	1.16
	(0.8–1.59)	(0.67–1.33)	(0.73–1.49)	(0.91–1.85)	(0.74–1.5)	(0.75–1.53)	(0.46–1.01)	(0.56–1.15)	(0.47–1.02)	(0.58–1.16)	(0.56–1.11)	(0.83–1.64)
**Dependent children**												
Yes	1 (referent)	1 (referent)	1 (referent)	1 (referent)	1 (referent)	1 (referent)	1 (referent)	1 (referent)	1 (referent)	1 (referent)	1 (referent)	1 (referent)
	p = 0.97	p = 0.15	p = 0.81	p = 0.94	p = 0.15	**p<0.0001**	p = 0.35	**p = 0.03**	**p = 0.02**	p = 0.67	p = 0.15	p = 0.10
No	1.00	1.15	0.94	1.03	1.15	0.59	0.90	0.80	0.77	0.96	1.15	1.18
	(0.83–1.22)	(0.95–1.4)	(0.77–1.14)	(0.84–1.25)	(0.95–1.4)	(0.49–0.72)	(0.73–1.12)	(0.65–0.97)	(0.62–0.96)	(0.79–1.17)	(0.95–1.4)	(0.97–1.43)
**State**												
ACT	1 (referent)	1 (referent)	1 (referent)	1 (referent)	1 (referent)	1 (referent)	1 (referent)	1 (referent)	1 (referent)	1 (referent)	1 (referent)	1 (referent)
	p = 0.39	p = 0.62	p = 0.13	p = 0.0722	p = 0.63	p = 0.96	p = 0.85	p = 0.38	p = 0.49	p = 0.28	p = 0.31	p = 0.83
NSW	0.90	1.41	1.14	1.75	1.63	1.14	1.00	1.21	1.23	1.55	1.34	0.88
	(0.5–1.65)	(0.77–2.56)	(0.61–2.12)	(0.89–3.44)	(0.89–2.97)	(0.61–2.12)	(0.51–1.97)	(0.62–2.33)	(0.6–2.53)	(0.82–2.95)	(0.73–2.46)	(0.48–1.59)
NT	0.74	5.06	4.04	2.67	2.45	1.08	2.38	2.40	2.38	5.02	2.07	1.18
	(0.18–3)	(0.94–27.16)	(0.89–18.27)	(0.65–11.01)	(0.54–11.02)	(0.26–4.52)	(0.57–10.01)	(0.59–9.83)	(0.55–10.31)	(1.11–22.68)	(0.5–8.57)	(0.29–4.89)
Queensland	1.14	1.37	1.20	1.97	1.68	1.07	1.01	1.21	1.17	1.57	1.28	0.99
	(0.62–2.11)	(0.74–2.51)	(0.64–2.27)	(0.99–3.9)	(0.91–3.09)	(0.57–2.02)	(0.5–2.01)	(0.62–2.37)	(0.56–2.43)	(0.81–3.02)	(0.69–2.38)	(0.54–1.82)
SA	0.89	1.24	1.35	2.12	1.83	0.93	0.92	1.38	1.61	1.76	1.40	0.82
	(0.47–1.7)	(0.65–2.36)	(0.69–2.63)	(1.04–4.32)	(0.96–3.51)	(0.48–1.82)	(0.44–1.91)	(0.68–2.78)	(0.75–3.45)	(0.89–3.49)	(0.73–2.68)	(0.43–1.56)
Tasmania	1.07	1.57	1.33	2.45	1.80	1.03	0.73	1.65	1.73	1.33	1.66	0.87
	(0.50–2.29)	(0.73–3.34)	(0.61–2.9)	(1.08–5.55)	(0.83–3.93)	(0.47–2.27)	(0.3–1.79)	(0.73–3.71)	(0.72–4.15)	(0.6–2.96)	(0.77–3.57)	(0.41–1.85)
Victoria	1.07	1.5	1.15	1.76	1.49	1.07	1.08	1.48	1.28	1.62	1.29	0.86
	(0.59–1.97)	(0.82–2.74)	(0.61–2.15)	(0.89–3.48)	(0.81–2.73)	(0.57–2.01)	(0.54–2.15)	(0.76–2.88)	(0.62–2.66)	(0.85–3.11)	(0.7–2.39)	(0.47–1.57)
WA	1.05	1.31	0.98	2.46	1.78	0.98	0.99	1.25	1.46	1.92	1.75	0.92
	(0.55–1.97)	(0.7–2.46)	(0.51–1.9)	(1.22–4.97)	(0.94–3.37)	(0.51–1.9)	(0.48–2.03)	(0.63–2.5)	(0.69–3.11)	(0.98–3.77)	(0.92–3.33)	(0.49–1.73)
unknown	1.31	1.5	1.53	1.81	1.43	1.11	1.06	1.04	1.44	1.27	1.04	1.16
	(0.65–2.64)	(0.75–3.01)	(0.75–3.14)	(0.84–3.89)	(0.71–2.88)	(0.54–2.29)	(0.48–2.34)	(0.49–2.24)	(0.63–3.28)	(0.6–2.66)	(0.51–2.11)	(0.58–2.33)
**Hosmer and Lemeshow Goodness of Fit test (p-value)**	p = 0.56	p = 0.62	p = 0.72	p = 0.64	p = 0.41	p = 0.64	p = 0.03	p = 0.51	p = 0.67	p = 0.66	p = 0.09	p = 0.67

^**1**^ The variable ‘cancer history with death’ pertains to cancer related deaths in close family members of the survey respondents.

**Abbreviation:** uni = university

Specifically, respondents with dependent children were significantly more likely to favour the funding for medicines for children (over adults), medicines for cancer diseases (over non-cancer diseases), and medicines for rare diseases (over common diseases) than those without children. Respondents who do not have private health insurance were significantly less likely to express a funding preference for treating patients whose diseases affect their ability to work (over those who are able to work despite their diseases) compared with those with private health insurance.

Respondents with a household income higher than $20,000 per year were more likely to express a preference for prioritising treatment of severe diseases (compared to moderate disease), treating patients for whom there are no alternative treatments available on the PBS instead of diseases for which several alternative treatments are available.

Respondents who are not in full time employment were more likely to favour treating patients who were not financially well-off (over those who are financially well-off patients), treating children (over adults), and treating life-style unrelated diseases (vs. life-style-related diseases). In addition, respondents aged 25 years or older were less likely to prioritise medicines for severe diseases (vs. moderate diseases), medicines for children (over adult patients), medicines for rare diseases (vs. common diseases) and ‘end-of-life treatments’.

In summary, all multivariate models satisfactorily fitted the data (p-value >0.05) except for ‘end-of-life treatment’ (p = 0.03), but the deviation between the observed and predicted outcomes of the model was minor.

## Discussion

Consideration of public preferences is desirable when making decisions about the funding of medicines given that the general public are both the payers and beneficiaries of any publicly funded health technologies [16, 26]. There is, therefore, an increasing recognition of the importance of taking into account public and patient preferences both in general and in relation to specific funding decisions [11]. Understanding what patients and the general public value about new medicines can improve alignment between government and societal preferences. This will, in turn, assist decision-makers to understand what societies are willing to support and forego in exchange for access to medicines [11].

The selection and reimbursement of prescribed medicines is inherently challenging and at times ethically controversial given the legislated requirement to consider the safety, efficacy, cost effectiveness and standard of manufacture of new medicines. This must be done using an evidence-based’ framework. In that context, where and how do public preferences/opinions fit into the decision making process? In Australia, the PBAC is not obliged to accept community preferences or opinions. But in seeking those very views the decision makers have an obligation to consider them in light of their charter to meet desired social objectives for the prescribed medicine budget. Inevitably that involves trade-offs and choices when considering the distribution of benefits and potential harms and costs of a particular decision. The key issue is that the whole process is informed by the best available information–including public preferences–and that there is transparent process for making an informed decision.

Under the assumption of ‘all else being equal’, this study suggests that severity of disease, diseases for which there is no alternative treatment available on the PBS (representing unmet need), diseases that affect patients who are not financially well off and life-style unrelated diseases are supported by the public as resource allocation criteria.

Further, contrary to some views [27–30] and somewhat surprising given the existence of “special funds” both in Australia and internationally for cancers and for rare diseases [31, 32], this study suggests that anti-cancer medicines and rare disease therapies per se are not factors that strongly drive public funding priorities. In fact, a large proportion of respondents favoured equal allocation of PBS money between (1) medicines for cancer vs. non cancer diseases (57.6%), and (2) medicines for rare vs. common diseases (42.6%). Notwithstanding the above, many new and expensive anti-cancer drugs are intended for rare cancers that are severe, life-threatening and for which there is no alternative treatment available on the PBS. Therefore, the public might nonetheless be supportive of resources being allocated to them.

When the assumption of treatment effectiveness or treatment costs are varied, it appears that allocation preferences are sensitive to both the health gains that may be realised and the number of patients who may benefit from a particular treatment. Under the health benefit trade-off condition, with the exception of ‘end-of-life treatment’, removing the assumption of equal treatment effectiveness generally led to a statistically significant shift in preferences towards the population that gained a considerable improvement in health and away from populations that gained a little improvement in health. Responses to cost (and corresponding number of patients treated) trade-off scenarios indicated a significant reduction in the proportion of respondents choosing to divide resources equally and a shift in preference towards devoting resources to the population that were more costly to treat for all criteria with the exception of severity of disease. The shift in respondents’ preferences to the populations that were more costly to treat may be driven by a reluctance to set priority based on cost, a concern with ensuring access to treatment based on need and/or a desire to not disadvantage patients with a high cost illness—even if this means that population health is not maximized [11, 17, 33, 34].

### Resonance with earlier studies

In line with the results of previous studies of public values [16, 17, 35, 36], this study provides evidence that members of the general public give higher priority to medicines used for the treatment of severe illness and for those with no available alternative, while no compelling evidence for prioritising ‘end-of-life treatments’ was observed. In the absence of other differences in patient or disease characteristics, or treatment effectiveness or costs, 49.7% of respondents divided resources evenly between ‘end-of-life therapies’ and ‘non end-of-life therapies’. However, previous studies suggested that the general public and patients with a life limiting illness expressed a preference/higher willingness to pay for treatments that could improve quality of life and value quality of care [20, 37, 38].

#### Comparison with the UK study by Linley et al 2013

Results for societal preferences for 8 of the 12 allocation criteria examined in this study were compared with the UK study by Linley et al [17]: (1) severity of disease, (2) availability of alternative medicine, (3) carer burden, (4) disadvantaged populations, (5) children, (6) ‘end-of-life treatments’, (7) cancer diseases, (8) rare disease therapies. In summary, there was a striking level of consistency between the views and preferences on allocation criteria in the general public of the UK and Australia.

Preferences under the assumption of ‘all else being equal’

Two of the three criteria identified by the UK participants as valid National Health Service (NHS) resource prioritisation criteria were supported by the Australian respondents. Both studies suggest, all else being equal, that severity of disease and disease for which no other available treatments exist are supported by society as valid NHS/PBS resource allocation criteria (disease severity: 59.6% and 52.7%; no other medicine available: 56.5% and 53.6% of respondents from the UK and Australian studies, respectively). Respondents in this study also expressed a preference for treating diseases that affect patients who are not financially well off (i.e. the disadvantaged populations) while the UK public supported prioritisation of medicines that reduce reliance on informal carers.

Preferences under health gain and cost trade-offs

The UK study did not include a benefit trade-off question relating to carer burden. Therefore, results relating to the benefit trade-off conditions for seven of the eight allocation criteria were compared. Similar to the UK general public [17], participants in this study expressed a shift in preferences towards the populations that gained a ‘considerable improvement in health’ and away from the populations that gained a ‘little health improvement’ with the exception of ‘end-of-life treatments’ when faced with health gain trade-offs.

Under the cost trade-off conditions, participants in this study and the UK study expressed a statistically significant shift in preferences towards the populations that were more costly to treat for all eight allocation criteria, with the exception of severity of disease.

### Implications for policy making

#### Implications for PBAC deliberations

The factors that are taken into consideration by the PBAC, as described in the 2013 PBAC guidelines [21], include readily quantifiable factors such as comparative cost effectiveness, comparative health gain, patient affordability in the absence of PBS subsidy, financial implications for the PBS and the Australian Government health budget, as well as less quantifiable factors such as uncertainty, equity, presence of effective alternatives, severity of medical condition treated, ability to target therapy with the proposed medicine precisely and effectively to patients likely to benefit most and development of resistance. Individual factors are not weighted equally by the PBAC in its decision making and the trade-offs involved in arriving at a recommendation, are not explicitly specified.

This study provides evidence of societal support for two of the PBAC decision criteria: disease severity and lack of alternative therapy for the medical condition being treated.

However, only 41% of respondents favoured prioritising patients whose out of pocket costs without PBS subsidy would be high compared to those whose out of pocket costs would be low.

In summary, the findings of this study suggest that the views of the Australian community are aligned with the PBAC when it comes to prioritising medicines that target severe diseases and/or for diseases for which there is no alternative treatment available on the PBS. However, ‘patient affordability in the absence of PBS subsidy’ may not be a shared prioritisation criterion between the PBAC and the general public.

#### Opportunity cost

The general public were less concerned about the opportunity cost of decisions (maximising population health), than they were about ensuring that resources are devoted to populations that are more costly to treat. This may be driven by concern for ensuring that patients whose diseases are expensive to treat are not disadvantaged, a desire to give all patients equal opportunity for access to treatment and/or a willingness to sacrifice health gains for a ‘fair’ public system over a single minded focus on efficiency of maximising population health [11, 17, 33, 34]. Given that cost to the PBS and government is one of the key criteria used in public funding decision for new medicines, this difference may explain the observed conflict between public and policy makers’ priorities when medicines are denied funding apparently on the basis of cost-ineffectiveness alone.

#### Rule of rescue criteria

The PBAC allows for consideration of ‘Rule of Rescue’ (RoR) criteria as part of its decision making process. A RoR applies in exceptional circumstances for pharmaceuticals that provide a worthwhile benefit for a severe and rare condition for which there is no alternative treatment [15, 21]. For drugs that meet the RoR criteria, the PBAC could potentially reverse a decision not to recommend listing on the basis of comparative cost-effectiveness (and any other relevant factors). This study explored three of the four criteria for PBS listing under the RoR, namely disease severity, lack of alternative treatment option and rarity of a disease [21]. Although disease severity and lack of alternative medicine for the medical condition were supported as allocation criteria by our participants, we observed no compelling evidence to support the rarity of disease criterion. In this study, only 26% of respondents favoured prioritising patients with a rare disease in the absence of any other differences.

#### Life saving drugs program criteria

Through its Life Saving Drugs Program (LSDP), the Australian Government provides subsidised access to expensive and life saving drugs that are not eligible for funding under the PBS, for very rare life-threatening conditions [32]. To receive LSDP funding, there are eight criteria that a drug must meet. This study explored three of the LSDP criteria: lack of alternative treatment options, rarity of a disease and affordability of the medicine. Although lack of alternative treatment option was supported in this study, the other two criteria (rarity of disease, patient affordability due to cost of the drugs) were not regarded as important in determining the distribution of subsidised PBS medicines by our respondents. This suggests that the use of rarity, and patient affordability as health technology assessment funding criteria for the LSDP appear to be open to question and require further scrutiny.

It is worth noting that the LSDP is currently under review by the Australian Government. The review examines issues such as access and equity, value for money and the future administration of the programme [39]. The public consultation/submission process for the LSDP review was closed in 2015. However, there is no timeframe specified for the outcome of the review.

### Strength and limitations

The strengths of this study were that it included a large, broadly representative sample (n = 3080) of the Australian population. The format adopted for eliciting preferences of the survey allowed an easy comparison of shift in preferences to provide a complete picture of respondent trade-off behaviours using either health gains or costs alone. The results of this study are consistent with other studies and notably a study by Linley et al [17], upon which this study was based.

This study has limitations. The main limitation is that we simplified the survey task for participants by varying one allocation criterion at a time. We did not ask the public to consider multiple allocation criteria simultaneously, as the PBAC must do for any given submission. Whilst this study allowed for the rank ordering of relative importance of each allocation criterion, no conclusions can be made about any interaction effects among criteria. As such, it would be useful to capture these complexities in future research. To minimise respondent burden and the number of criteria explored in this study, we also did not include all of the criteria considered by the PBAC for PBS and LSDP listing. Due to the study design, details for non responders were not available for analysis or assessment for potential bias.

Another potential limitation relates to framing bias. The questions in this study were framed to encourage expressions of societal preferences for the distribution of prescribed medicines. We did not seek individual’s views on direct questions of opportunity cost–a concept operationalised by the use of cost effectiveness information by the expert government committee. It is also possible that respondents’ own interpretations of the allocation scenarios have the potential to influence their expressed preferences.

The results of this study suggest that respondent preferences may be influenced by their personal circumstances. While some of these relationships have clear and plausible explanations, some are more difficult to explain. For example, relationship observed for respondents without private health insurance and their expressed preferences for lifestyle unrelated diseases.

### Implications for future research

Understanding and incorporation of public preferences and public engagement in public finding allocation for medicines is an important step towards ensuring the legitimacy, relevance and fairness of decision making and might reduce conflicts between public and payers regarding public funding allocation [11, 35, 40]. The results of this study give a clear picture of public preferences regarding medicines resource allocation and demonstrate that the general public are capable of giving opinions on distributional preferences. To enable effective integration of public and patient preferences into funding decisions, further research on defining a strategy to incorporate public perspectives into PBAC decision making processes is required.

## Conclusion

Given that decisions about funding of new medicines have a direct impact on the general public through cost and access constraints [26], it is important that these decisions/decision making process take into account societal preferences and the community’s willingness to pay alongside the needs of the patients. Knowledge of public preferences and values allow policy makers to better understand the societal issues of importance and has the potential to reduce conflicts between public and payers regarding public funding allocation [11, 35, 40].

Bodies such as the UK’s NICE and Australia’s PBAC have the expertise and resources to assess questions of comparative clinical benefit, cost, safety and quality of manufacture. They are also well-placed to consider the opportunity cost of funding prescribed medicines. But it is the general public who are best placed to consider societal views on the fairness of those decisions. By any measure, almost all organised effort is expended in assessing the efficiency of funding decisions for prescribed medicines. Comparatively little effort is expended in considering the distributional consequences of expert committee recommendations. A person-centered approach to health care implies that we ask the public how they want spending decisions to reflect their preferences for the distribution of benefits and costs of prescribed medicines. Therefore, if there is a commitment that public preferences matter, then it would be important for decision makers to consider and incorporate the public perspectives as part of the funding decision making process.

## Supporting information

S1 File Questionnaire for Cohort 1. (PDF)

S2 File Questionnaire for Cohort 2. (PDF)
