# Supplementary material for: Societal perspective on access to publicly subsidised medicines: A cross sectional survey of 3080 adults in Australia
Source: PLoS One. 2017 Mar 1;12(3):e0172971. doi: 10.1371/journal.pone.0172971 (PMC5332102; doi:10.1371/journal.pone.0172971)
Supplement: S2 File — (PDF) [file pone.0172971.s002.pdf]

Societal perspective on access to publicly subsidised medicines: A cross sectional survey of 3080 adults in Australia

Web based survey (Cohort 2)

Who should get access to Government subsidised prescribed medicines? Have your say.

Introduction:
For over 70 years the Australian Government has provided its people with access to safe and affordable prescribed medicines through a scheme known as the Pharmaceutical Benefits Scheme (PBS). This means that the price you pay for many prescribed medicines is limited to a certain amount (co-payment) – currently $37.70 for general beneficiaries and $ 6.10 if you have a concession card.

To ensure that government money is spent wisely, the PBS requires evidence that a medicine is sufficiently safe, can be manufactured to a high standard, provides health benefits and is value for money.

One important consideration in how PBS money is spent is "fairness". Who should get access to subsidised medicines? Is one group of patients more deserving than another? That's where your views are important.

This survey is designed to find out what you think is 'fair" in terms of how the PBS might best spend government money.

In the following questions you will be presented with hypothetical scenarios. Each question is designed to get your opinion on YOUR preferred way for the PBS to spend public money. Please read these carefully and indicate your preferred way for the PBS to spend money. There are no right or wrong answers – it's a matter of opinion—and we want to know what YOU think.

Scenario 1: Severity of disease

What's Fair? Should more PBS money go to patients with **severe health problems** compared to those with **moderate health problems?**

Imagine that there are two diseases that are identical in every respect except that:

- One causes **severe health problems**
- The other causes **moderate health problems**.

Where would you like to see the PBS spend its money – more towards patients with severe health problems or those with moderate health problems?

In the table below you can allocate PBS money for 100 patients, identical in every respect except for their underlying health problems. There are different combinations of patients with severe compared to moderate health problems.

Each combination adds up to 100 patients. Where would you allocate the money?

Please indicate your preference by ticking the combination that best reflects your opinion on 'what's fair'.

	All money spent on the disease that causes **severe health problems**					Money divided equally					All money spent on the disease that causes **moderate health problems**
	Treatment for **100** patients with severe health problems	Treatment for **90** patients with severe health problems	Treatment for **80** patients with severe health problems	Treatment for **70** patients with severe health problems	Treatment for **60** patients with severe health problems	Treatment for **50** patients with severe health problems	Treatment for **40** patients with severe health problems	Treatment for **30** patients with severe health problems	Treatment for **20** patients with severe health problems	Treatment for **10** patients with severe health problems	Treatment for **0** patients with severe health problems
	And **0** patients with moderate health problems	And **10** patients with moderate health problems	And **20** patients with moderate health problems	And **30** patients with moderate health problems	And **40** patients with moderate health problems	And **50** patients with moderate health problems	And **60** patients with moderate health problems	And **70** patients with moderate health problems	And **80** patients with moderate health problems	And **90** patients with moderate health problems	And **100** patients with moderate health problems
Tick one box	☐	☐	☐	☐	☐	☐	☐	☐	☐	☐	☐

Scenario 1: Severity of disease (Part A2: Cost trade-off question)

In the previous question, the same medicine was used to treat the disease that causes severe health problems, and the disease that causes moderate health problems.

Now imagine that there are two different medicines, Medicine A and Medicine B. Medicine A is used to treat the disease that causes severe health problems, while Medicine B is used to treat the disease that causes moderate health problems.

Medicines A and B are equally safe and effective, but differ in terms of **how much they cost the PBS and, therefore, how many patients can be treated.**

The PBS is now able to pay for treatment for a maximum of:

- **50 patients** with **severe health problems** (treated using the more expensive Medicine A)
- **100 patients** with **moderate health problems** (treated using the less expensive Medicine B).

In the table below you can allocate PBS money for up to 100 patients. There are different combinations of treating fewer patients with severe health problems, and treating more patients with moderate health problems. Where would you allocate the money?

Please indicate your preference by ticking the combination that best reflects your opinion on ‘what’s fair’.

		All money spent on Medicine A: the **more expensive medicine** that is used to treat patients with **severe health problems**					Money divided equally					All money spent on Medicine B: the **less expensive medicine** that is used to treat patients with **moderate health problems**
	Total number of patients treated	50	55	60	65	70	75	80	85	90	95	100
	Number of patients with severe health problems (treated with Medicine A)	50	45	40	35	30	25	20	15	10	5	0
	Number of patients with moderate health problems (treated with medicine B)	0	10	20	30	40	50	60	70	80	90	100
Tick one box		☐	☐	☐	☐	☐	☐	☐	☐	☐	☐	☐

Scenario 2: Availability of alternative treatment options

What's Fair? Should more PBS money go to patients for whom there are **no alternative treatments available on the PBS** compared to those for whom there are **several alternative treatments already available on the PBS?**

Imagine that there are two diseases that are identical in every respect except that:

- For one disease, there is **only one treatment available** on the PBS
- For the other disease, there are **several alternative treatments** available on the PBS

Where would you like to see the PBS spend its money – more towards patients for whom there are no alternative treatments available on the PBS, or those for whom there are several alternative treatments already available on the PBS?

In the table below you can allocate PBS money for 100 patients, identical in every respect except for how many different treatments are available. There are different combinations of patients for whom there is only one treatment available on the PBS and those for whom there are several alternative treatments already available on the PBS.

Each combination adds up to 100 patients. Where would you allocate the money?

Please indicate your preference by ticking the combination that best reflects your opinion on 'what's fair'.

	All money spent on the disease for which **there is only this one treatment**					Money divided equally					All money spent on the disease for which **there are several alternative treatments**
	Treatment for **100** patients for whom there are no alternative treatments	Treatment for **90** patients for whom there are no alternative treatments	Treatment for **80** patients for whom there are no alternative treatments	Treatment for **70** patients for whom there are no alternative treatments	Treatment for **60** patients for whom there are no alternative treatments	Treatment for **50** patients for whom there are no alternative treatments	Treatment for **40** patients for whom there are no alternative treatments	Treatment for **30** patients for whom there are no alternative treatments	Treatment for **20** patients for whom there are no alternative treatments	Treatment for **10** patients for whom there are no alternative treatments	Treatment for **0** patients for whom there are no alternative treatments
	And **0** patients for whom there are several alternative treatments	And **10** patients for whom there are several alternative treatments	And **20** patients for whom there are several alternative treatments	And **30** patients for whom there are several alternative treatments	And **40** patients for whom there are several alternative treatments	And **50** patients for whom there are several alternative treatments	And **60** patients for whom there are several alternative treatments	And **70** patients for whom there are several alternative treatments	And **80** patients for whom there are several alternative treatments	And **90** patients for whom there are several alternative treatments	And 1**00** patients for whom there are several alternative treatments
Tick one box	☐	☐	☐	☐	☐	☐	☐	☐	☐	☐	☐

Scenario 2: Availability of alternative treatment options (Part A2: Cost trade-off question)

In the previous question, the same medicine was used to treat the disease for which there is only one available treatment, and the disease for which there are several available alternative treatments.

Now imagine that there are two different medicines, Medicine A and Medicine B. Medicine A is used to treat the disease for which there is only one available treatment (which is Medicine A), while Medicine B is used to treat the disease for which there are several available alternative treatments, including but not limited to Medicine B.

Medicines A and B are equally safe and effective, but differ in terms of **how much they cost the PBS and, therefore, how many patients can be treated.**

The PBS is now able to pay for treatment for a maximum of:

- **50 patients** for whom there is **only one treatment available** (treated using the more expensive Medicine A)
- **100 patients** for whom there are **several alternative treatments** (treated using the less expensive Medicine B).

In the table below you can allocate PBS money for up to 100 patients. There are different combinations of treating fewer patients for whom there is only one treatment available, and treating more patients for whom there are several alternative treatments. Where would you allocate the money?

Please indicate your preference by ticking the combination that best reflects your opinion on 'what's fair'.

		All money spent on Medicine A: the **more expensive medicine** that is used to treat patients for whom there is **only one treatment available**					Money divided equally					All money spent on Medicine B: the **less expensive medicine** that is used to treat patients for whom there are **several alternative treatments available**
	Total number of patients treated	50	55	60	65	70	75	80	85	90	95	100
	Number of patients for whom there is only one treatment available (treated with Medicine A)	50	45	40	35	30	25	20	15	10	5	0
	Number of patients for whom there are several treatment alternatives (treated with medicine B)	0	10	20	30	40	50	60	70	80	90	100
Tick one box		☐	☐	☐	☐	☐	☐	☐	☐	☐	☐	☐

Scenario 3: Innovative medicine

What's Fair? Should more PBS money go to treatments that **work in new ways** compared to treatments that **work the same way as existing treatments?**

Imagine that there are two diseases that are identical in every respect except that:

- For one disease, the treatment that is available is innovative— that is, it has a mechanism of action that is unlike any other existing medicine
- For the other disease, the treatment that is available is not innovative—that is, it works in the same way as several other existing medicines.

The two treatments are equally safe and effective.

Where would you like to see the PBS spend its money – more towards patients who have a disease that has an innovative treatment, or those for whom the treatment is not innovative?

In the table below you can allocate PBS money for 100 patients, identical in every respect except for whether or not their disease has an innovative treatment. There are different combinations of patients for whom there is an innovative treatment and those for whom there is a non-innovative treatment.

Each combination adds up to 100 patients. Where would you allocate the money?

Please indicate your preference by ticking the combination that best reflects your opinion on 'what's fair'.

	All money spent on the patients who would receive the medicine that has a **new mechanism of action (is innovative)**					Money divided equally					All money spent on the patients who would receive the medicine that **works in the same way as other existing medicines (is not innovative)**
	Treatment for **100** patients who would receive the innovative treatment	Treatment for **90** patients who would receive the innovative treatment	Treatment for **80** patients who would receive the innovative treatment	Treatment for **70** patients who would receive the innovative treatment	Treatment for **60** patients who would receive the innovative treatment	Treatment for **50** patients who would receive the innovative treatment	Treatment for **40** patients who would receive the innovative treatment	Treatment for **30** patients who would receive the innovative treatment	Treatment for **20** patients who would receive the innovative treatment	Treatment for **10** patients who would receive the innovative treatment	Treatment for **0** patients with who would receive the innovative treatment
	And **0** patients who would receive the non-innovative treatment	And **10** patients who would receive the non-innovative treatment	And **20** patients who would receive the non-innovative treatment	And **30** patients who would receive the non-innovative treatment	And **40** patients who would receive the non-innovative treatment	And **50** patients who would receive the non-innovative treatment	And **60** patients who would receive the non-innovative treatment	And **70** patients who would receive the non-innovative treatment	And **80** patients who would receive the non-innovative treatment	And **90** patients who would receive the non-innovative treatment	And **100** patients who would receive the non-innovative treatment
Tick one box	☐	☐	☐	☐	☐	☐	☐	☐	☐	☐	☐

Scenario 3: Innovative medicine (Part A2: Cost trade-off question)

In the previous question, the only difference between the two medicines used to treat the two diseases was that one medicine was innovative and one was not innovative.

Now imagine that the two medicines differ also in terms of **how much they cost the PBS and, therefore, how many patients can be treated.**

The PBS is now able to pay for treatment for a maximum of:

- **50 patients** using the more expensive medicine (A), which **is the innovative medicine** (has a novel mechanism of action)
- **100 patients** using the less expensive medicine (B), which **is not innovative** (works the same way as other, existing medicines).

In the table below you can allocate PBS money for up to 100 patients. There are different combinations of treating fewer patients with the innovative medicine, and treating more patients with the non-innovative medicine. Where would you allocate the money?

Please indicate your preference by ticking the combination that best reflects your opinion on 'what's fair'.

		All money spent on <u>Medicine A:</u> the **more expensive and innovative medicine**					Money divided equally					All money spent on <u>Medicine B:</u> the **less expensive and non-innovative medicine**
	Total number of patients treated	50	55	60	65	70	75	80	85	90	95	100
	Number of patients treated with the innovative medicine (Medicine A)	50	45	40	35	30	25	20	15	10	5	0
	Number of patients treated with the non-innovative medicine (Medicine B)	0	10	20	30	40	50	60	70	80	90	100
Tick one box		☐	☐	☐	☐	☐	☐	☐	☐	☐	☐	☐

Scenario 4: Carer burden

What's Fair? Should more PBS money go to patients **who have to rely on carers** compared to those **who do not have to rely on carers**?

Imagine that there are two diseases that are identical in every respect except that:

- One causes patients to depend on carers (e.g. family members) for their day-to-day needs
- The other does not cause patients to depend on carers—i.e. patients are unwell, but they remain independent in fulfilling their day-to-day needs.

Where would you like to see the PBS spend its money – more towards patients who rely on carers or those who remain independent?

In the table below you can allocate PBS money for 100 patients, identical in every respect except for whether or not their disease causes them to rely on carers. There are different combinations of patients who rely on carers compared to those who remain independent.

Each combination adds up to 100 patients. Where would you allocate the money?

Please indicate your preference by ticking the combination that best reflects your opinion on 'what's fair'.

	All money spent on the patients who **rely on carers for their day-to-day needs**					Money divided equally					All money spent on the patients who **remain independent in fulfilling their day-to-day needs.**
	Treatment for **100** patients who rely on carers	Treatment for **90** patients who rely on carers	Treatment for **80** patients who rely on carers	Treatment for **70** patients who rely on carers	Treatment for **60** patients who rely on carers	Treatment for **50** patients who rely on carers	Treatment for **40** patients who rely on carers	Treatment for **30** patients who rely on carers	Treatment for **20** patients who rely on carers	Treatment for **10** patients who rely on carers	Treatment for **0** patients who rely on carers
	And **0** patients who do not rely on carers	And **10** patients who do not rely on carers	And **20** patients who do not rely on carers	And **30** patients who do not rely on carers	And **40** patients who do not rely on carers	And **50** patients who do not rely on carers	And **60** patients who do not rely on carers	And **70** patients who do not rely on carers	And **80** patients who do not rely on carers	And **90** patients who do not rely on carers	And **100** patients who do not rely on carers
Tick one box	☐	☐	☐	☐	☐	☐	☐	☐	☐	☐	☐

Scenario 4: Carer burden (Part A2: Cost trade-off question)

In the previous question, the same medicine was used to treat the disease that causes patients to depend on carers and the disease that allows patients to remain independent.

Now imagine that there are two different medicines, Medicine A and Medicine B. Medicine A is used to treat the disease that makes people dependent on carers, while Medicine B is used to treat the disease that does not cause dependence on carers.

Medicines A and B are equally safe and effective, but differ in terms of **how much they cost the PBS and, therefore, how many patients can be treated.**

The PBS is now able to pay for treatment for a maximum of:

- **50 patients** who are **dependent on carers** (treated using the more expensive Medicine A)
- **100 patients** who **remain independent** (treated using the less expensive Medicine B)**.**

In the table below you can allocate PBS money for up to 100 patients. There are different combinations of treating fewer patients who are dependent on carers, and treating more patients who are independent. Where would you allocate the money?

Please indicate your preference by ticking the combination that best reflects your opinion on 'what's fair'.

		All money spent on <u>Medicine A:</u> the **more expensive medicine** that is used to treat patients who are **dependent on carers for their day-to-day needs**					Money divided equally					All money spent on <u>Medicine B:</u> the **less expensive medicine** that is used to treat patients who **remain independent in fulfilling their day-to-day needs**
	Total number of patients treated	50	55	60	65	70	75	80	85	90	95	100
	Number of patients who are dependent on cares (treated with Medicine A)	50	45	40	35	30	25	20	15	10	5	0
	Number of patients who are independent (treated with medicine B)	0	10	20	30	40	50	60	70	80	90	100
Tick one box		☐	☐	☐	☐	☐	☐	☐	☐	☐	☐	☐

Scenario 5: Patient income

What's Fair? Should more PBS money go to patients who are **financially well-off** compared to those who are **not financially well-off?**

Imagine that there are two diseases that are identical in every respect except that:

- One typically affects **patients who are not financially well-off,** e.g. those from low income families
- The other typically affects patients who are **financially well-off** e.g. those from families with good incomes.

Where would you like to see the PBS spend its money – more towards patients who are not financially well-off or those who are financially well-off?

In the table below you can allocate PBS money for 100 patients, identical in every respect except for their level of financial security. There are different combinations of patients who are not financially well-off compared to those who are financially well-off.

Each combination adds up to 100 patients. Where would you allocate the money?

Please indicate your preference by ticking the combination that best reflects your opinion on 'what's fair'.

	All money spent on the disease that affects patients who are not **financially well off**					Money divided equally					All money spent on the disease that affects patients who are **financially well off**
	Treatment for **100** patients who are not well off	Treatment for **90** patients who are not well off	Treatment for **80** patients who are not well off	Treatment for **70** patients who are not well off	Treatment for **60** patients who are not well off	Treatment for **50** patients who are not well off	Treatment for **40** patients who are not well off	Treatment for **30** patients who are not well off	Treatment for **20** patients who are not well off	Treatment for **10** patients who are not well off	Treatment for **0** patients who are not well off
	And **0** patients who are well off	And **10** patients who are well off	And **20** patients who are well off	And **30** patients who are well off	And **40** patients who are well off	And **50** patients who are well off	And **60** patients who are well off	And **70** patients who are well off	And **80** patients who are well off	And **90** patients who are well off	And **100** patients who are well off
Tick one box	☐	☐	☐	☐	☐	☐	☐	☐	☐	☐	☐

Scenario 5: Patient income (Part A2: Cost trade-off question)

In the previous question, the same medicine was used to treat the disease that affects those who are financially well-off, and the disease that affects those who are not well-off.

Now imagine that there are two different medicines, Medicine A and Medicine B. Medicine A is used to treat the disease that affects those who not well-off, while Medicine B is used to treat the disease that affects those who are well-off.

Medicines A and B are equally safe and effective, but differ in terms of **how much they cost the PBS and, therefore, how many patients can be treated.**

The PBS is now able to pay for treatment for a maximum of:

- **50 patients** who are **not well off financially** (treated using the more expensive Medicine A)
- **100 patients** who are **well off financially** (treated using the less expensive Medicine B)**.**

In the table below you can allocate PBS money for up to 100 patients. There are different combinations of treating fewer patients who are not well-off, and treating more patients who are well-off. Where would you allocate the money?

Please indicate your preference by ticking the combination that best reflects your opinion on ‘what’s fair’.

		All money spent on <u>Medicine A:</u> the **more expensive medicine** that is used to treat patients who are **not well off financially**					Money divided equally					All money spent on <u>Medicine B:</u> the **less expensive medicine** that is used to treat patients who are **well off financially**
	Total number of patients treated	50	55	60	65	70	75	80	85	90	95	100
	Number of patients who are not well off (treated with Medicine A)	50	45	40	35	30	25	20	15	10	5	0
	Number of patients who are well off (treated with medicine B)	0	10	20	30	40	50	60	70	80	90	100
Tick one box		☐	☐	☐	☐	☐	☐	☐	☐	☐	☐	☐

Scenario 6: Patient age

What's Fair? Should more PBS money go to **treating children** compared to **treating adults?**

Imagine that there are two diseases that are identical in every respect except that:

- One typically **affects children**
- The other typically **affects adults.**

Where would you like to see the PBS spend its money – more towards treating children, or more towards treating adults?

In the table below you can allocate PBS money for 100 patients, identical in every respect except for their age. There are different combinations of children compared to adults.

Each combination adds up to 100 patients. Where would you allocate the money?

Please indicate your preference by ticking the combination that best reflects your opinion on 'what's fair'.

	All money spent on the disease that **affects children**					Money divided equally					All money spent on the disease that **affects adults**
	Treatment for **100** children	Treatment for **90** children	Treatment for **80** children	Treatment for **70** children	Treatment for **60** children	Treatment for **50** children	Treatment for **40** children	Treatment for **30** children	Treatment for **20** children	Treatment for **10** children	Treatment for **0** children
	And **0** adults	And **10** Adults	And **20** adults	And **30** adults	And **40** adults	And **50** adults	And **60** adults	And **70** adults	And **80** adults	And **90** adults	And **100** adults
Tick one box	☐	☐	☐	☐	☐	☐	☐	☐	☐	☐	☐

Scenario 6: Patient age (Part A2: Cost trade-off question)

In the previous question, the same medicine was used to treat the disease that affects children and the disease that affects adults.

Now imagine that there are two different medicines, Medicine A and Medicine B. Medicine A is used to treat the disease that affects children, while Medicine B is used to treat the disease that affects adults.

Medicines A and B are equally safe and effective, but differ in terms of **how much they cost the PBS and, therefore, how many patients can be treated.**

The PBS is now able to pay for treatment for a maximum of:

- **50 children** (using the more expensive Medicine A)
- **100 adults** (using the less expensive Medicine B)**.**

In the table below you can allocate PBS money for up to 100 patients. There are different combinations of treating fewer children, and treating more adults. Where would you allocate the money?

Please indicate your preference by ticking the combination that best reflects your opinion on ‘what’s fair’.

		All money spent on Medicine A: the **more expensive medicine** that is used to treat **children**					Money divided equally					All money spent on Medicine B: the **less expensive medicine** that is used to treat **adults**
	Total number of patients treated	50	55	60	65	70	75	80	85	90	95	100
	Number of children (treated with Medicine A)	50	45	40	35	30	25	20	15	10	5	0
	Number of adults (treated with medicine B)	0	10	20	30	40	50	60	70	80	90	100
Tick one box		☐	☐	☐	☐	☐	☐	☐	☐	☐	☐	☐

Scenario 7: Life expectancy

What's Fair? Should more PBS money go to patients who would **die within 18 months** without treatment compared to those who would **die within 60 months** without treatment?

Imagine that there are two diseases that are **both fatal**, and are identical in every respect except that, without treatment:

- One causes patients to die within 18 months of diagnosis (within one and a half years)
- The other causes patients to die within 60 months of diagnosis (within five years).

Where would you like to see the PBS spend its money – more towards patients who would die within 18 months, or those who would die within 60 months?

In the table below you can allocate PBS money for 100 patients, identical in every respect except for their underlying health problems. There are different combinations of patients who would die within 18 months compared with those who would die within 60 months.

Each combination adds up to 100 patients. Where would you allocate the money?

Please indicate your preference by ticking the combination that best reflects your opinion on 'what's fair'.

	All money spent on patients who **would die within 18 months**					Money divided equally					All money spent on patients who **would die within 60 months**
	Treatment for **100** patients who would die within 18 months	Treatment for **90** patients who would die within 18 months	Treatment for **80** patients who would die within 18 months	Treatment for **70** patients who would die within 18 months	Treatment for **60** patients who would die within 18 months	Treatment for **50** patients who would die within 18 months	Treatment for **40** patients who would die within 18 months	Treatment for **30** patients who would die within 18 months	Treatment for **20** patients who would die within 18 months	Treatment for **10** patients who would die within 18 months	Treatment for **0** patients who would die within 18 months
	And 0 patients who would die within 60 months	And **10** patients who would die within 60 months	And **20** patients who would die within 60 months	And **30** patients who would die within 60 months	And **40** patients who would die within 60 months	And **50** patients who would die within 60 months	And **60** patients who would die within 60 months	And **70** patients who would die within 60 months	And **80** patients who would die within 60 months	And **90** patients who would die within 60 months	And **100** patients who would die within 60 months
Tick one box	☐	☐	☐	☐	☐	☐	☐	☐	☐	☐	☐

Scenario 7: Life expectancy (Part A2: Cost trade-off question)

In the previous question, the same medicine was used to treat the disease that, if untreated, causes death within 18 months (1.5 years) and the disease that, if untreated, causes death within 60 months (5 years).

Now imagine that there are two different medicines, Medicine A and Medicine B. Medicine A is used to treat the disease that causes death within 18 months, while Medicine B is used to treat the disease that causes death within 60 months.

Medicines A and B are equally safe and effective, but differ in terms of **how much they cost the PBS and, therefore, how many patients can be treated.**

The PBS is now able to pay for treatment for a maximum of:

- **50 patients** who would **die within 18 months** without treatment (treated using the more expensive Medicine A)
- **100 patients** who would **die within 60 months** without treatment (treated using the less expensive Medicine B).

In the table below you can allocate PBS money for up to 100 patients. There are different combinations of treating fewer patients who would die within 18 months, and treating more patients who would die within 60 months. Where would you allocate the money?

Please indicate your preference by ticking the combination that best reflects your opinion on 'what's fair'.

		All money spent on Medicine A: the **more expensive medicine** that is used to treat patients who would **die within 18 months without treatment**					Money divided equally					All money spent on Medicine B: the **less expensive medicine** that is used to treat patients who would **die within 60 months without treatment**
	Total number of patients treated	50	55	60	65	70	75	80	85	90	95	100
	Number of patients who would die within 18 months (treated with Medicine A)	50	45	40	35	30	25	20	15	10	5	0
	Number of patients who would die within 60 months (treated with medicine B)	0	10	20	30	40	50	60	70	80	90	100
Tick one box		☐	☐	☐	☐	☐	☐	☐	☐	☐	☐	☐

Scenario 8: Cancer vs. non-cancer disease

What's Fair? Should more PBS money go to patients **who have cancer** compared to those who have a **disease that is not cancer?**

Imagine that there are two diseases that are identical in every respect except that:

- One is a type of cancer
- The other is a non-cancer type of disease.

Where would you like to see the PBS spend its money – more towards patients with cancer or those with non-cancer types of diseases?

In the table below you can allocate PBS money for 100 patients, identical in every respect except for their underlying health problems. There are different combinations of patients with cancer compared to non-cancer types of disease.

Each combination adds up to 100 patients. Where would you allocate the money?

Please indicate your preference by ticking the combination that best reflects your opinion on 'what's fair'.

	All money spent on the medicine used to treat **cancer patients**					Money divided equally					All money spent on the medicine used to treat patients with a **disease that is not cancer**
	Treatment for **100** patients with cancer	Treatment for **90** patients with cancer	Treatment for **80** patients with cancer	Treatment for **70** patients with cancer	Treatment for **60** patients with cancer	Treatment for **50** patients with cancer	Treatment for **40** patients with cancer	Treatment for **30** patients with cancer	Treatment for **20** patients with cancer	Treatment for **10** patients with cancer	Treatment for **0** patients with cancer
	And **0** patients with a non-cancer disease	And **10** patients with a non-cancer disease	And **20** patients with a non-cancer disease	And **30** patients with a non-cancer disease	And **40** patients with a non-cancer disease	And **50** patients with a non-cancer disease	And **60** patients with a non-cancer disease	And **70** patients with a non-cancer disease	And **80** patients with a non-cancer disease	And **90** patients with a non-cancer disease	And **100** patients with a non-cancer disease
Tick one box	☐	☐	☐	☐	☐	☐	☐	☐	☐	☐	☐

Scenario 8: Cancer vs. non-cancer disease (Part A2: Cost trade-off question)

In the previous question, the same medicine was used to treat the cancer and the non-cancer disease.

Now imagine that there are two different medicines, Medicine A and Medicine B. Medicine A is used to treat the cancer, while Medicine B is used to treat non-cancer disease.

Medicines A and B are equally safe and effective, but differ in terms of **how much they cost the PBS and, therefore, how many patients can be treated.**

The PBS is now able to pay for treatment for a maximum of:

- **50 patients** with **cancer** (treated using the more expensive Medicine A)
- **100 patients** with **the non-cancer disease** (treated using the less expensive Medicine B).

In the table below you can allocate PBS money for up to 100 patients. There are different combinations of treating fewer patients with cancer, and treating more patients with the non-cancer disease. Where would you allocate the money?

Please indicate your preference by ticking the combination that best reflects your opinion on ‘what’s fair’.

		All money spent on <u>Medicine A:</u> the **more expensive medicine** that is used to treat patients with **cancer**					Money divided equally					All money spent on <u>Medicine B:</u> the **less expensive medicine** that is used to treat patients with **the non-cancer disease**
	Total number of patients treated	50	55	60	65	70	75	80	85	90	95	100
	Number of patients with cancer (treated with Medicine A)	50	45	40	35	30	25	20	15	10	5	0
	Number of patients with non-cancer disease (treated with medicine B)	0	10	20	30	40	50	60	70	80	90	100
Tick one box		☐	☐	☐	☐	☐	☐	☐	☐	☐	☐	☐

Scenario 9: Common vs. rare diseases

What's Fair? Should more PBS money go to patients with **rare diseases** compared to those with **common diseases?**

Imagine that there are two diseases that are identical in every respect except that:

- One is rare (affects less than 2000 patients in Australia), and
- The other is common (affects more than 500,000 patients in Australia).

Where would you like to see the PBS spend its money – more towards patients with rare diseases or those with common diseases?

In the table below you can allocate PBS money for 100 patients, identical in every respect except for their underlying health problems. There are different combinations of patients with rare compared to common diseases.

Each combination adds up to 100 patients. Where would you allocate the money?

Please indicate your preference by ticking the combination that best reflects your opinion on 'what's fair'.

	All money spent on the medicine used to treat the **rare disease**					Money divided equally					All money spent on the medicine used to treat the **common disease**
	Treatment for<br>**100**<br>patients with the rare disease	Treatment for<br>**90**<br>patients with the rare disease	Treatment for<br>**80**<br>patients with the rare disease	Treatment for<br>**70**<br>patients with the rare disease	Treatment for<br>**60**<br>patients with the rare disease	Treatment for<br>**50**<br>patients with the rare disease	Treatment for<br>**40**<br>patients with the rare disease	Treatment for<br>**30**<br>patients with the rare disease	Treatment for<br>**20**<br>patients with the rare disease	Treatment for<br>**10**<br>patients with the rare disease	Treatment for<br>**0**<br>patients with the rare disease
	And<br>**0**<br>patients with the common disease	And<br>**10**<br>patients with the common disease	And<br>**20**<br>patients with the common disease	And<br>**30**<br>patients with the common disease	And<br>**40**<br>patients with the common disease	And<br>**50**<br>patients with the common disease	And<br>**60**<br>patients with the common disease	And<br>**70**<br>patients with the common disease	And<br>**80**<br>patients with the common disease	And<br>**90**<br>patients with the common disease	And<br>**100**<br>patients with the common disease
Tick one box	☐	☐	☐	☐	☐	☐	☐	☐	☐	☐	☐

Scenario 9: Common vs. rare disease (Part A2: Cost trade-off question)

In the previous question, the same medicine was used to treat the rare disease (that affects less than 2000 Australians) and the common disease (that affects more than 500,000 Australians).

Now imagine that there are two different medicines, Medicine A and Medicine B. Medicine A is used to treat the rare disease, while Medicine B is used to treat the common disease.

Medicines A and B are equally safe and effective, but differ in terms of **how much they cost the PBS and, therefore, how many patients can be treated.**

The PBS is now able to pay for treatment for a maximum of:

- **50 patients** with the **rare disease** (treated using the more expensive Medicine A)
- **100 patients** with the **common disease** (treated using the less expensive Medicine B).

In the table below you can allocate PBS money for up to 100 patients. There are different combinations of treating fewer patients with the rare disease, and treating more patients with the common disease. Where would you allocate the money?

Please indicate your preference by ticking the combination that best reflects your opinion on 'what's fair'.

		All money spent on Medicine A: the **more expensive medicine** that is used to treat patients with **the rare disease**					Money divided equally					All money spent on Medicine B: the **less expensive medicine** that is used to treat patients with **the common disease**
	Total number of patients treated	50	55	60	65	70	75	80	85	90	95	100
	Number of patients with the rare disease (treated with Medicine A)	50	45	40	35	30	25	20	15	10	5	0
	Number of patients with the common disease (treated with medicine B)	0	10	20	30	40	50	60	70	80	90	100
Tick one box		☐	☐	☐	☐	☐	☐	☐	☐	☐	☐	☐

Scenario 10: Cost to the PBS and savings to patients

What's Fair? Should more PBS money go to patients **whose out of pocket costs without PBS subsidy would be high** compared to those whose **out of pocket costs would be low?**

Imagine that there are two diseases that are identical in every respect except that:

- For one disease, the treatment costs the PBS $5000 per month to subsidise and saves patients $4960 (assuming a $40 co-payment)
- For the other disease, the treatment costs the PBS $100 per month to subsidise and saves patients $60 (assuming the same $40 co-payment).

The two treatments are equally safe and effective.

Where would you like to see the PBS spend its money – more towards patients who have a disease that costs the PBS $5000/saves patients $4960 per month
or those who have the disease that costs the PBS $100/saves patients $60 per month.

In the table below you can allocate PBS money for 100 patients, identical in every respect except for how much their treatment costs the PBS. There are different combinations of patients whose treatment costs the PBS $5000 per month (saving patients $4960) and those whose treatment costs the PBS $100 per month (saving patients $60).

Each combination adds up to 100 patients. Where would you allocate the money?

Please indicate your preference by ticking the combination that best reflects your opinion on 'what's fair'.

	All money spent on the medicine that costs the PBS $5000 per month to subsidise and saves patients $4960					Money divided equally					All money spent on the medicine that costs the PBS $100 per month to subsidise and saves patients $60
	Treatment for<br>**100**<br>patients who need the medicine that costs the PBS $5000/saves patients $4960 per month	Treatment for<br>**90**<br>patients who need the medicine that costs the PBS $5000/saves patients $4960 per month	Treatment for<br>**80**<br>patients who need the medicine that costs the PBS $5000/saves patients $4960 per month	Treatment for<br>**70**<br>patients who need the medicine that costs the PBS $5000/saves patients $4960 per month	Treatment for<br>**60**<br>patients who need the medicine that costs the PBS $5000/saves patients $4960 per month	Treatment for<br>**50**<br>patients who need the medicine that costs the PBS $5000/saves patients $4960 per month	Treatment for<br>**40**<br>patients who need the medicine that costs the PBS $5000/saves patients $4960 per month	Treatment for<br>**30**<br>patients who need the medicine that costs the PBS $5000/saves patients $4960 per month	Treatment for<br>**20**<br>patients who need the medicine that costs the PBS $5000/saves patients $4960 per month	Treatment for<br>**10**<br>patients who need the medicine that costs the PBS $5000/saves patients $4960 per month	Treatment for<br>**0**<br>patients who need the medicine that costs the PBS $5000/saves patients $4960 per month
	And<br>**0**<br>patients who need the medicine that costs the PBS $100/saves patients $60 per month	And<br>**10**<br>patients who need the medicine that costs the PBS $100/saves patients $60 per month	And<br>**20**<br>patients who need the medicine that costs the PBS $100/saves patients $60 per month	And<br>**30**<br>patients who need the medicine that costs the PBS $100/saves patients $60 per month	And<br>**40**<br>patients who need the medicine that costs the PBS $100/saves patients $60 per month	And<br>**50**<br>patients who need the medicine that costs the PBS $100/saves patients $60 per month	And<br>**60**<br>patients who need the medicine that costs the PBS $100/saves patients $60 per month	And<br>**70**<br>patients who need the medicine that costs the PBS $100/saves patients $60 per month	And<br>**80**<br>patients who need the medicine that costs the PBS $100/saves patients $60 per month	And<br>**90**<br>patients who need the medicine that costs the PBS $100/saves patients $60 per month	And<br>**100**<br>patients who need the medicine that costs the PBS $100/saves patients $60 per month

Tick one box	☐	☐	☐	☐	☐	☐	☐	☐	☐	☐	☐

No cohort 2 for scenario 10: Cost to PBS and patients

Scenario 11: Ability to work

What's Fair? Should more PBS money go to patients **whose diseases affect their ability to work** compared to those **who are able to continue working despite their disease?**

Imagine that there are two diseases that are identical in every respect except that:

- One typically impacts upon patients' ability to work (i.e. patients typically need to quit work because of their disease)
- The other does not impact upon patients' ability to work.

Where would you like to see the PBS spend its money – more towards patients who cannot work without treatment or those who can continue to work despite their disease?

In the table below you can allocate PBS money for 100 patients, identical in every respect except for their underlying health problems. There are different combinations of patients who cannot work without treatment and those who can continue to work despite their disease.

Each combination adds up to 100 patients. Where would you allocate the money?

Please indicate your preference by ticking the combination that best reflects your opinion on 'what's fair'.

	All money spent on the disease that **prevents patients from working**					Money divided equally					All money spent on the disease that **does not prevent patients from working**
	Treatment for **100** patients who cannot work	Treatment for **90** patients who cannot work	Treatment for **80** patients who cannot work	Treatment for **70** patients who cannot work	Treatment for **60** patients who cannot work	Treatment for **50** patients who cannot work	Treatment for **40** patients who cannot work	Treatment for **30** patients who cannot work	Treatment for **20** patients who cannot work	Treatment for **10** patients who cannot work	Treatment for **0** patients who cannot work
	And **0** patients who are able to work	And **10** patients who are able to work	And **20** patients who are able to work	And **30** patients who are able to work	And **40** patients who are able to work	And **50** patients who are able to work	And **60** patients who are able to work	And **70** patients who are able to work	And **80** patients who are able to work	And **90** patients who are able to work	And **100** patients who are able to work
Tick one box	☐	☐	☐	☐	☐	☐	☐	☐	☐	☐	☐

Scenario 11: Ability to work (Part A2: Cost trade-off question)

In the previous question, the same medicine was used to treat the disease that prevents people from working and the disease that does not prevent people from working.

Now imagine that there are two different medicines, Medicine A and Medicine B. Medicine A is used to treat the disease that prevents people from working, while Medicine B is used to treat the disease that does not prevent people from working.

Medicines A and B are equally safe and effective, but differ in terms of **how much they cost the PBS and, therefore, how many patients can be treated.**

The PBS is now able to pay for treatment for a maximum of:

- **50 patients who are unable to work** as a result of their disease (treated using the more expensive Medicine A)
- **100 patients** who are **still able to work** despite their disease (treated using the less expensive Medicine B).

In the table below you can allocate PBS money for up to 100 patients. There are different combinations of treating fewer patients who are unable to work because of their disease, and treating more who are able to work despite their disease. Where would you allocate the money?

Please indicate your preference by ticking the combination that best reflects your opinion on 'what's fair'.

		All money spent on Medicine A: the **more expensive medicine** that is used to treat patients who are **unable to work because of their disease**					Money divided equally					All money spent on Medicine B: the **less expensive medicine** that is used to treat patients who are **still able to work despite their disease**
	Total number of patients treated	50	55	60	65	70	75	80	85	90	95	100
	Number of patients who are unable to work (treated with Medicine A)	50	45	40	35	30	25	20	15	10	5	0
	Number of patients who are able to work (treated with medicine B)	0	10	20	30	40	50	60	70	80	90	100
Tick one box		☐	☐	☐	☐	☐	☐	☐	☐	☐	☐	☐

Scenario 12: Lifestyle-related disease

What's Fair? Should more PBS money go to patients with **lifestyle-related diseases** compared to those with **diseases that are not related to lifestyle?**

Imagine that there are two diseases that are identical in every respect except that:

- One is not considered to be a lifestyle-related disease (i.e. it could not be avoided through lifestyle-related choices such as diet or exercise)
- One is considered to be a lifestyle-related disease (e.g. disease secondary to obesity or smoking).

Where would you like to see the PBS spend its money – more towards patients whose disease is not related to lifestyle or those who have lifestyle-related disease.

In the table below you can allocate PBS money for 100 patients, identical in every respect except for their underlying health problems. There are different combinations of patients with the non lifestyle-related disease compared to the lifestyle-related disease.

Each combination adds up to 100 patients. Where would you allocate the money?

Please indicate your preference by ticking the combination that best reflects your opinion on 'what's fair'.

	All money spent on the disease that is **unrelated to lifestyle**					Money divided equally					All money spent on the disease that is **related to lifestyle**
	Treatment for **100** patients with a disease unrelated to lifestyle	Treatment for **90** patients with a disease unrelated to lifestyle	Treatment for **80** patients with a disease unrelated to lifestyle	Treatment for **70** patients with a disease unrelated to lifestyle	Treatment for **60** patients with a disease unrelated to lifestyle	Treatment for **50** patients with a disease unrelated to lifestyle	Treatment for **40** patients with a disease unrelated to lifestyle	Treatment for **30** patients with a disease unrelated to lifestyle	Treatment for **20** patients with a disease unrelated to lifestyle	Treatment for **10** patients with a disease unrelated to lifestyle	Treatment for **0** patients with disease a unrelated to lifestyle
	And **0** patients with lifestyle-related disease	And **10** patients with lifestyle-related disease	And **20** patients with lifestyle-related disease	And **30** patients with lifestyle-related disease	And **40** patients with lifestyle-related disease	And **50** patients with lifestyle-related disease	And **60** patients with lifestyle-related disease	And **70** patients with lifestyle-related disease	And **80** patients with lifestyle-related disease	And **90** patients with lifestyle-related disease	And **100** patients with lifestyle-related disease
Tick one box	☐	☐	☐	☐	☐	☐	☐	☐	☐	☐	☐

Scenario 12: Lifestyle-related disease (Part A2: Cost trade-off question)

In the previous question, the same medicine was used to treat the disease that is not related to lifestyle and the disease that is lifestyle-related.

Now imagine that there are two different medicines, Medicine A and Medicine B. Medicine A is used to treat the disease that is not related to lifestyle, while Medicine B is used to treat the disease that is lifestyle-related.

Medicines A and B are equally safe and effective, but differ in terms of **how much they cost the PBS and, therefore, how many patients can be treated.**

The PBS is now able to pay for treatment for a maximum of:

- **50 patients** with the disease that is **not related to lifestyle** (treated using the more expensive Medicine A)
- **100 patients** with the **lifestyle-related disease** (treated using the less expensive Medicine B).

In the table below you can allocate PBS money for up to 100 patients. There are different combinations of treating fewer patients with non lifestyle-related problems, and treating more patients with lifestyle-related problems. Where would you allocate the money?

Please indicate your preference by ticking the combination that best reflects your opinion on 'what's fair'.

		All money spent on <u>Medicine A:</u> the **more expensive medicine** that is used to treat patients with a **non lifestyle-related disease**					Money divided equally					All money spent on <u>Medicine B:</u> the **less expensive medicine** that is used to treat patients with a **lifestyle-related disease**
	Total number of patients treated	50	55	60	65	70	75	80	85	90	95	100
	Number of patients with a non lifestyle-related disease (treated with Medicine A)	50	45	40	35	30	25	20	15	10	5	0
	Number of patients with a lifestyle-related disease (treated with medicine B)	0	10	20	30	40	50	60	70	80	90	100
Tick one box		☐	☐	☐	☐	☐	☐	☐	☐	☐	☐	☐
